# Neurocomputational modeling of rule abstraction and memorization during probabilistic stimulus-reward learning

**DOI:** 10.1016/j.isci.2025.113950

**Published:** 2025-11-14

**Authors:** René Schlegelmilch, Alina Dinu, Gina Joue, Jan Gläscher, Tobias Sommer

**Affiliations:** 1 Department of Psychology, University of Bremen, Bremen, Germany; 2 Institute of Systems Neuroscience, University Medical Center Hamburg-Eppendorf, Hamburg, Germany

**Keywords:** cognitive neuroscience and psychology

## Abstract

Preferential choice among multi-attribute stimuli commonly involves one of the two learning strategies: rule abstraction and memorization. When stimulus features combine in a systematic, albeit complex way to predict rewarding or punishing outcomes (e.g., a combination of color and shape distinguishes edible from poisonous mushrooms), corresponding learning problems can be solved via rule abstraction. In other problems lacking this systematicity, stimuli have to be memorized individually. Here, we use fMRI, eye-tracking, and cognitive modeling to study how humans deploy these two learning strategies to select between two simultaneously presented objects. We observed differential learning trajectories and fixation patterns, indicating sudden rule discovery and incremental learning, respectively, captured by cognitive modeling. The derived process estimates allowed us to identify overlapping brain networks associated with cognitive control and value-based decision-making. Importantly, our multi-modal data and model-informed analyses link those processes to unique brain regions, revealing the neurocognitive mechanisms of rule abstraction and memorization.

## Highlights

• Value-based choice uses rule abstraction or memorization of stimuli
• Rule learning entails sudden insight, while memorization shows gradual learning
• The CAL model captures both via behavior and eye-tracking attention
• FMRI links each strategy to distinct control, decision, and memory networks

## Graphical abstract

**Figure:**
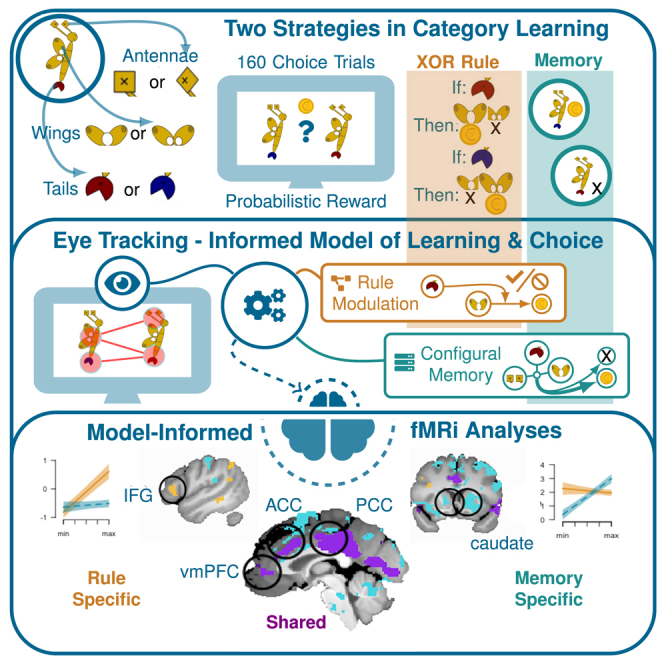

## Introduction

Our everyday life is filled with preferential choices among multi-attribute stimuli. An example of this situation is the choice among two restaurants, which can be described on several different dimensions, such as nationality, interior style, size (number of tables), and time since it opened. The decision problem can be solved by two fundamentally different strategies: a decision rule or by remembering past experiences, which both evolve from prior learning. When learning to abstract decision rules, our aim is to identify relevant dimensions and among them systematic combinations that predict rewarding experiences. For instance, we might observe that nationality A and traditional style or nationality B and modern style lead to satisfying experiences. This strategy can only be successful when such a rule exists. Searching for a perfectly predictive rule implies many failed attempts until the rule is discovered, after which performance stays at near perfect levels.^1^

In contrast, memorization relies on the encoding of idiosyncratic stimulus representations and their associated rewards. These memories are retrieved and compared with the current stimulus to inform future decisions.^2^ However, the need to maintain distinct memory traces for potentially very similar stimuli implies a more gradual learning trajectory until strong configural memory representations are established.^3^^,^^4^ Importantly, those processing differences not only concern learning trajectories but also distinct patterns of attention allocation. While rule abstraction entails focusing attention on relevant dimensions (feature attention), memorization requires holistic attention to the stimulus.^5^^,^^6^

In this study, we aim to characterize the neurocomputational signature of these two different learning strategies using a unified computational framework. In this framework, we jointly model behavior, process-level data (eye-tracking), and neural data to identify the brain networks they recruit. Such a direct comparison of these opposing learning strategies at the computational and neural levels is currently lacking in the literature. Two of the six classic category learning problems, Type II and Type IV, provide an ideal test-bed for investigating these learning strategies under controlled experimental conditions.^7^^,^^8^^,^^9^

**Figure 1:**
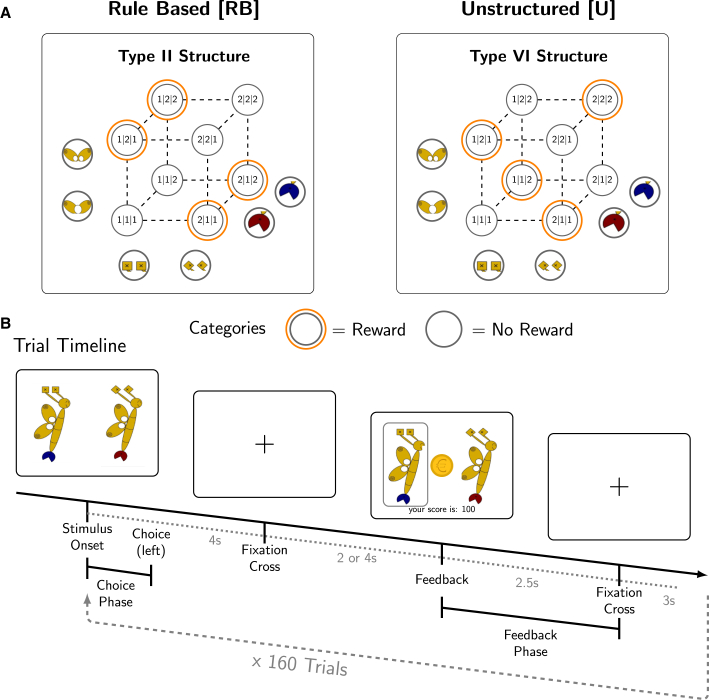
Task design and procedure (A) Rule based [RB] and unstructured [U] learning problems. Numbers = coordinates of corresponding binary features (wings, antennae, tail color). Orange circles = reward category. (B) 44 Participants learned RB and U, using butterfly or humanoid stimuli, respectively (counterbalanced). They received instructions on the rule strategy before the RB and the memory strategy in U. (Stimulus Onset). They were instructed to choose the rewarding stimulus. Feedback was probabilistic and in 80% correct.

Type II problems implement a disjunctive stimulus-outcome relation (XOR), in which the opposing features of two dimensions belong to the same category. For example, traditional and nationality A or modern and nationality B are categorized as tasty, while traditional and nationality B or modern and nationality A are not particularly appealing (see Figure 1A). This disjunctive problem is emblematic of rule abstraction, henceforth referred to as “rule-based” (RB). In contrast, none of these systematic contingencies exist in Type VI problems, which are further referred to as “unstructured” (U).^10^ Solving this type of problem requires memorizing each individual stimulus. Importantly, our goal is to investigate these strategies in the context of value-based decision-making. We therefore adapted the classic “sorting stimuli into categories” paradigm so that participants had to choose between two multi-attribute stimuli to receive probabilistic reward feedback (see Figure 1B).

We deployed the recently developed category abstraction learning (CAL) model,^11^ which explicitly distinguishes between rule abstraction and memorization within its cognitive architecture. CAL extrapolates first-order contingencies – termed Simple Rules – on independent stimulus dimensions (e.g., color predicts outcomes), updating these rules through similarity-based generalization and dissimilarity-based contrasting.^12^^,^^13^^,^^14^ Crucially, CAL combines this Simple Rule learning with error-driven modulation,^15^^,^^16^^,^^17^ implemented as a recency-weighted tally of rule successes and errors, enabling context-dependent response gating.^18^^,^^19^ This mechanism predicts sudden, step-like improvements in performance when a rule is discovered, as previously observed in Type II category-learning problems.^11^^,^^20^ In contrast, when no consistent rule can be abstracted (as in Type VI problems), CAL relies on a configural-memory module that gradually strengthens associations between individual stimulus configurations and outcomes. By jointly modeling rule extrapolation, modulation, and memorization, CAL accounts for a variety of distinct behavioral signatures at both the group and individual level, including process measures such as eye movements, in several classic and probabilistic learning tasks.^5^^,^^7^^,^^9^^,^^11^^,^^21^ This makes it an ideal candidate for estimating learning processes in our study, in which we adapted the model to stimulus selection with probabilistic feedback. We summarize its central hypotheses in the STAR Methods and provide full formal details in the supplemental information.

We expected to find unique brain regions supporting the two opposing strategies because they presume fundamentally different cognitive processes. We hypothesized that the inferior frontal cortex (IFG) may be recruited for rule abstraction, while the posterior hippocampus may be involved in memorization. However, we also anticipated the presence of commonly activated brain networks associated with both learning strategies due to shared cognitive processes involved in attentional control and value-based decision-making. Previous research suggests that the caudate and ventromedial prefrontal cortex (vmPFC) play roles in these processes.^1^^,^^22^^,^^23^^,^^24^^,^^25^^,^^26^^,^^27^^,^^28^^,^^29^^,^^30^ It should be noted, though, that memorization in this context does not necessarily imply declarative memory processes and hippocampal involvement,^31^ as exemplar processing has also been related to striatal processes.^32^ We show that CAL was capable of modeling specific characteristics of the behavioral and process data of our participants, further used in model-informed fMRI analyses. Those revealed a common network supporting preferential choice in both types of problems, including regions in the posterior hippocampus and vmPFC, middle frontal and supramarginal gyrus (MFG, SFG), anterior and posterior cingulate cortex (ACC, PCC), as well as the N. accumbens. However, they also revealed unique networks in RB and U, respectively, including the IFG and the caudate.

### Rule-based and unstructured learning task

We exposed 44 participants to both rule-based (RB) and unstructured (U) problems using comparable discrete-feature stimuli. Figure 1 illustrates the corresponding abstract problem representations used in previous categorization literature.^7^^,^^33^ In our study, we replaced traditional^3^^,^^7^^,^^9^^,^^11^^,^^34^^,^^35^^,^^36^ category labels (e.g., A versus B) with categorical reward associations (reward versus no reward), highlighting the conceptual overlap between category learning and value-based decision-making with multidimensional, discrete-feature stimuli.^37^^,^^38^^,^^39^ The example shown in Figure 1 illustrates one of the two stimulus sets used in the experiment, varying between RB and U within participants (butterflies or humanoids, see STAR Methods).

The stimuli comprised three visual dimensions (antennae, wings, and tail), with binary features (e.g., square or diamond antenna). In each of the 160 trials, participants decided which of the two stimuli was associated with a monetary reward, receiving feedback corresponding to the respective stimulus-reward associations. The feedback was probabilistic, with the true reward stimulus yielding a reward in 80% of the trials. Furthermore, in the RB condition, participants could learn that one dimension was irrelevant (e.g., tails in Figure 1A; counterbalanced between participants), while in the U condition, they had to memorize the stimuli to obtain rewards.

The diverse nature of neural processes within Type II learning has been studied before, typically in comparison to simpler rule-based tasks.^22^^,^^26^^,^^40^^,^^41^ However, cognitive studies documented the heterogeneity of learning strategies in Type II rule-learning tasks,^11^^,^^17^^,^^21^^,^^42^^,^^43^^,^^44^^,^^45^^,^^46^ which could complicate the comparison of strategy-specific learning processes.^47^^,^^48^ To address this, we homogenized participants’ learning by explicitly instructing them on the optimal strategy for solving the tasks – namely, a disjunctive two-dimensional rule without revealing which specific rule, or through stimulus memorization, for the RB and U conditions, respectively. Additionally, we trained participants on these strategies using different stimuli and deterministic feedback, and we familiarized them with probabilistic feedback before they began the main learning task, which was conducted in the fMRI scanner.

## Results

### Strategies differ on learning trajectories, but not on accuracy

**Figure 2:**
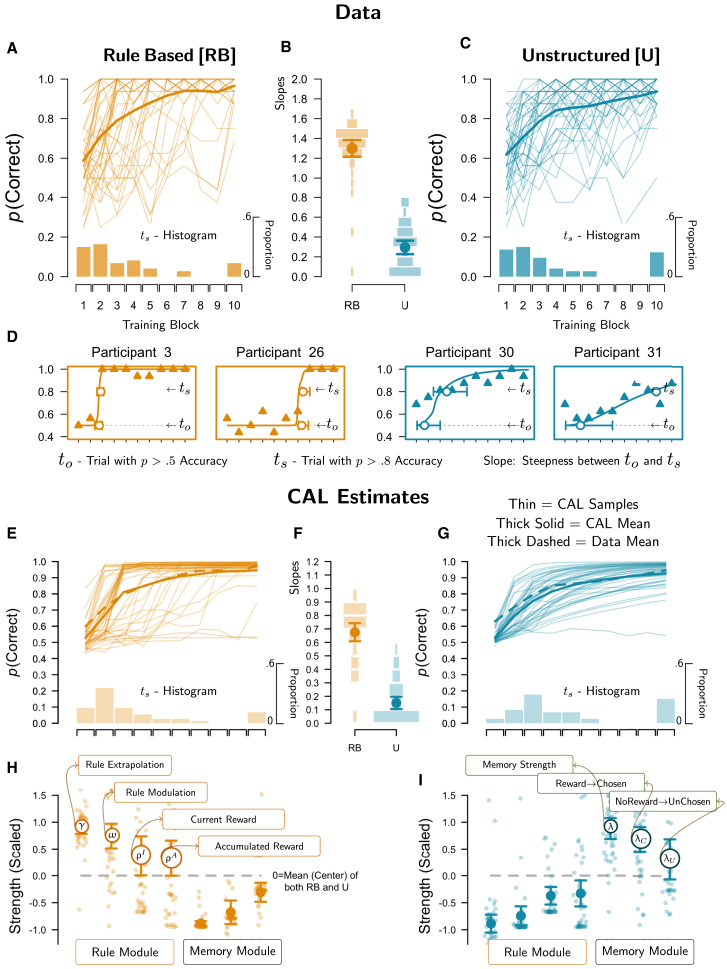
Behavioral data and CAL estimates (A and C) Canonical accuracy (thick line = average, thin lines = individuals, *N* = 44) for each RB and U over training (16 trial blocks). The lower *t*_*s*_ histograms show the distribution of individually estimated trials, in which accuracy exceeded 80% (*t*_*s*_ = trial solved); the value in block 10 indicates the proportion of participants never reaching 80% accuracy or only in that block. (B) Means (symbols) and individual (histograms) learning-slope estimates (y axis; higher values = steeper slopes). (D) highlights characteristic individual trajectories. Lines indicate logistic regression fits (symbols = mean participant data), measuring the onset trial of learning (*t*_*o*_), the trial in which accuracy reached 80% (*t*_*s*_), and the slope of accuracy between these points (shown in B). (E–G) Parallel to (A, B, C) for CAL. (E and G) shows CAL’s learning curves after model fitting (dashed line = mean data; thick solid = mean CAL for comparison). The *t*_*s*_ histograms and slopes in (F) were derived from logistic regression applied to CAL’s learning curves, as obtained for participant data. (H and I) show CAL’s cognitive parameter estimates in RB and U, respectively, z-scored (mean = 0, SD = 1) for visual comparison between tasks (higher values indicate a greater contribution relative to the other task, highlighting distinct module contributions; see STAR Methods for parameter overview). Error bars represent 95% confidence intervals.

The average forward learning curves were nearly identical between both tasks (Figures 2A and 2B; thick lines). However, it is known that average forward curves disguise strategy-relevant differences, and corresponding discussions favor methods disclosing when and how quickly participants transition from chance-like performance to a perfect solution, such as backward learning curves.^49^^,^^50^ Especially the backward curve in Type II dissociates humans from non-human performance.^20^ Indeed, our individual learning trajectories (thin lines in Figures 2A and 2B) unveiled that participants in RB solved the task suddenly after numerous trials of chance-like performance, suggesting spontaneous rule discovery, while in U, individual accuracy changed gradually, indicating the incremental memorization of the stimuli. Instead of backward learning curves, however, we applied a modified hierarchical Bayesian logistic regression to derive each individual’s trial, in which performance first reliably differs from guessing (*t*_o_), the trial in which the task was solved (*t*_s_), and consequently, the individual’s slope in between both (steepness of transition; see further STAR Methods). Consequently, we compare behavior depending on *t*_s_ as “Before Solution” versus “After Solution.” as similarly done in previous research.^5^

First, the individual posteriors for *t*_s_ confirmed that participants solved both tasks equally quickly, based on a paired *t* test using the R package BayesFactor, *M*_Diff_ = −8.59, *SD* = 69.49, *d* = −0.13, 95CI [-0.41, 0.16], BF_10_ = 0.24.^51^ However, the learning slopes (log scale) were substantially steeper in RB than in U, *M*_Diff_ = 0.99, *SD* = 0.35, *d* = 2.88, 95CI [2.21, 3.58], BF_10_ > 100 (Figure 2C), translating to an average difference between estimated guessing and solution trials of *M* = 5.34 trials in RB, and *M* = 28.14 trials in U. After participants solved the task, the probabilistic feedback hardly changed their behavior, suggesting error discounting.

### Category abstraction learning captures task-dependent differential learning trajectories

Figures 2E–2G shows that CAL accurately captured these ordinal effects in individual learning curves (thin lines) and average learning curves (thick solid lines), via rule discovery in RB (discovered rule modulation), but a more gradual increase in U (Hebbian strengthening of exemplar associations).^52^ As recommended for model evaluation^53^^,^^54^ we obtained CAL’s corresponding learning Slope estimates, by applying the same logistic regression to the CAL fits as to the participants before, to see whether we obtained the same results (posterior predictive check). Indeed, we observed the same ordinal main effect between RB and U in a Bayesian *t* test, *M*_Diff_ = 0.52, *SD* = 0.28, *d* = 1.86, 95CI [1.36, 2.38], BF_10_ > 100, corroborated by very strong correlations between CAL’s *t*_*s*_ estimates and those of the participants, yielding *r* = 0.97, BF > 1000, 95%HDI[0.94, 0.98] in RB, and *r* = 0.67, BF > 1000, 95%HDI[0.48, 0.80] in U.

### Learning strategies affect feature attention but not stimulus attention

**Figure 3:**
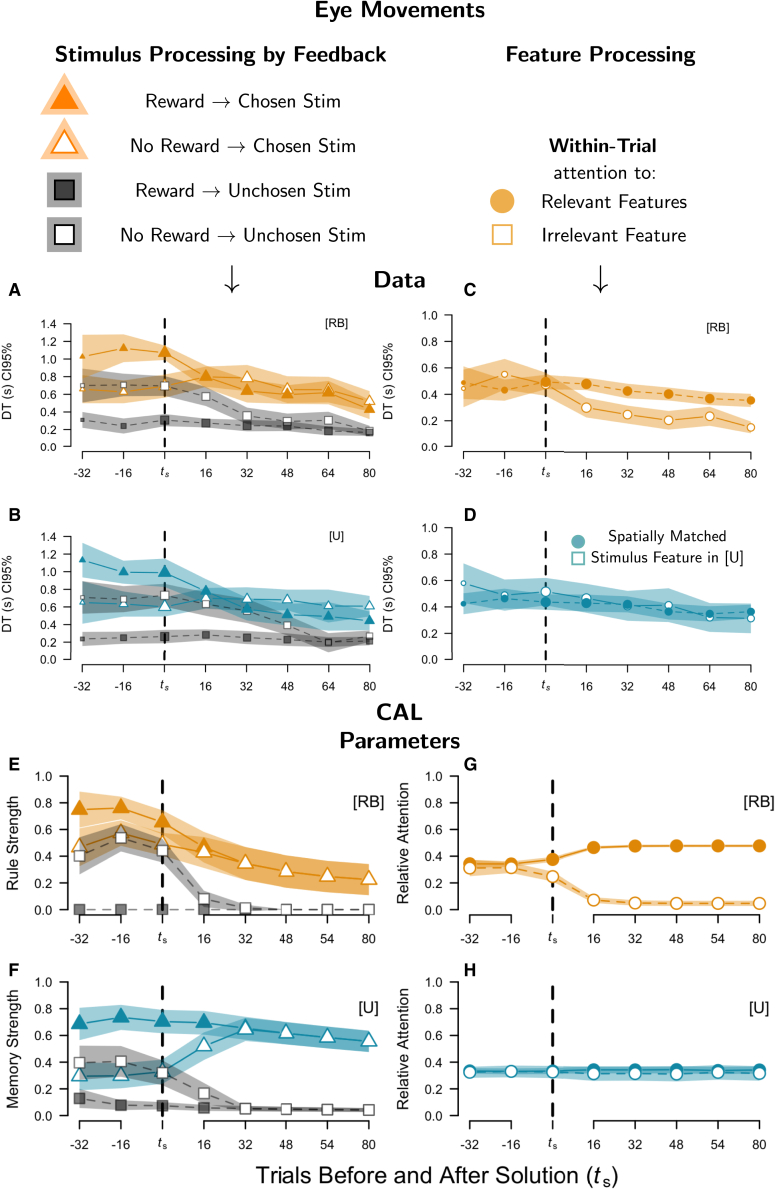
Stimulus (left) and feature attention (right) and CAL process estimates (A–D) Mean dwell times (DT in seconds) relative to the trial, in which participants solved the task (*t*_*s*_). (A and B) DT on chosen versus unchosen stimuli over training periods, modulated by received feedback between trials (attention to: chosen after reward [orange, filled triangle], chosen after no reward [orange, hollow triangle], unchosen after reward [gray, filled square], unchosen after no reward [gray, hollow square]). (C and D) For the RB task, DTs of the two relevant features (filled symbols) were averaged and compared to the irrelevant feature (hollow symbol). For the U task, all features were relevant; to enable comparison regarding the decline in attention on the irrelevant RB feature, features in U were grouped by spatial location, matching the irrelevant feature’s position in RB (e.g., if “wings” was irrelevant in RB, the hollow symbol in U represents DTs to the “wings” feature). CAL parameters (E–H) in analogue display for Stimulus and Feature attention. (E and F) Stimulus processing, as used in fMRI is CAL-informed processing strength (RB: *v*_*t*_; U: *p*(C—S), see STAR Methods). In RB, higher strength = stronger simple rule extrapolation, lower values = stronger rule modulation. In U, higher values = stronger Hebbian update. (G and H) CAL’s relative feature attention (α and β trial-wise averaged, then calculated as for the participants’ feature attention; see STAR Methods for details). All error bars/shadings = CI95%.

Figures 3A–3D indicate a significant drop in stimulus and feature processing when solving the task (post *t*_*s*_). Overall, the results are consistent with rule abstraction in RB, in which the relevant dimensions are identified and then, within them, the reward-predicting features are attended to. This also translates to corresponding variations in response times in the choice phase (not shown). A mixed-effects model analysis of response times^55^ (see STAR Methods) revealed significant main effects of block (coded relative to *t*_*s*_), *χ*^2^(7) = 371.20, *p* < 0.001, task, *χ*^2^(1) = 14.61, *p* < 0.001, and an interaction between both, *χ*^2^(7) = 78.96, *p* < 0.001. In post-hoc tests^56^ with Bonferroni correction for 8 block-wise tests, there was no difference in RTs in block 0 (containing *t*_*s*_), *M*_*Diff*_ = 0.020, *SE* = 0.062, *p* = 1 or earlier (all *p* >0.25), while the difference was significant in all blocks >0 (all *p* < 0.01; e.g., block 1, *M*_*Diff*_ = −0.3357, *SE* = 0.062, *p* < 0.001). Further analyses of the eye-tracking data unveiled a corresponding effect in overall dwell times (DTs), further indicating that participants paid more attention to the chosen relative to the unchosen stimulus (not shown), which is termed the gaze-cascade effect in preferential decision-making^57^^,^^58^ (Figure S3). However, the reduction in DTs seemed mainly driven by selective feature attention in RB relative to U (analyzed later in discussion; Figures 3B–3D).

In the outcome phase, when participants received feedback, however, we found effects shared between tasks regarding stimulus attention (dwell times, DTs) depending on the valence of the reward feedback (reward vs. no-reward trials). Figures 3A and 3B show that before the solution, the chosen stimulus received by far the most attention after reward feedback, while the unchosen stimulus was rather ignored. In no-reward trials, both stimuli, on average, received a relatively equal amount of attention. After the solution, however, participants only attended to the chosen stimulus regardless of the feedback, again suggesting error discounting. In a corresponding mixed model, we found significant main effects of stimulus, *χ*^2^(1) = 62.74, *p* <0.001, block, *χ*^2^(1) = 329.15, *p* <0.001, and reward, *χ*^2^(1) = 32.10, *p* <0.001. We further observed a two-way interaction between stimulus and reward, *χ*^2^(1) = 7.22, *p* = 0.007, and three-way interaction with block, *χ*^2^(7) = 239.22, *p* <0.001, reflecting the trends in Figures 3A and 3B. The only minor influence of task seemed to concern a two-way interaction between task and stimulus, *χ*^2^(1) = 7.22, *p* = 0.007, reflecting that the difference between the DTs on the chosen and unchosen stimuli were slightly more pronounced in RB than in U (the marginal difference in DT between chosen and in RB versus U was *M*_*Diff*_ = 0.0695, *p* = 0.009).

Crucially, task-specific attentional processing of the stimulus features further corroborated the conclusion of strategy-specific learning processes. Figures 2C and 2D (choice and outcome phases are collapsed in Figures 2A and 2B due to absent interactions) show that participants in RB ignored the irrelevant dimension after solving the task in both choice and outcome processing, as also reported in previous research^5^ speaking for rule discovery. For the analysis, we derived the summed DTs on each feature in a given trial and phase for both left and right stimuli. We then averaged the DTs of the two relevant features in that trial in RB, and did the same for the spatially matched features in U to highlight the difference in DTs between relevant versus irrelevant features and between both tasks (e.g., if the irrelevant feature in RB was the head, then we compared the DT to that for the head in U). The corresponding mixed model, including choice and outcome phases (trial phase), revealed significant main effects of task, *χ*^2^(1) = 4.19, *p* = 0.041, block, *χ*^2^(7) = 177.83, *p* <0.001, and trial phase, *χ*^2^(1) = 17.75, *p* <0.001. The latter indicates shorter DTs in outcome compared to choice, mainly due to the restricted time window during the former (see Figure 1B).

Crucially, there was a significant interaction between task and feature, *χ*^2^(1) = 18.71, *p* <0.001, and a three-way interaction with block, *χ*^2^(7) = 20.62, *p* = 0.004, reflecting the described trends (Figures 3B and 3D), which did not further interact with trial phase (choice vs. outcome), despite being somewhat less pronounced in outcome processing, *χ*^2^(7) = 1.99, *p* = 0.960. Additionally, there was an interaction between task and trial phase, *χ*^2^(1) = 21.25, *p* <0.001, and a three-way interaction with feature, such that the difference between choice and outcome processing was larger in RB than in U. All other effects were non-significant (all *p* >0.05).

In sum, while reward-dependent stimulus attention seemed to concern task-general processes changing between pre- and post-solution trials, the eye-tracking data regarding feature attention are consistent with the differential learning trajectories (sudden vs. incremental solution) and our optimal-strategy instructions in each RB and U, respectively.

### Category abstraction learning replicates attentional processing in multi-measure comparison

Figures 2H and 2I further show the individual and mean estimates for CAL’s free parameters. All of its rule learning parameters were stronger in RB than in U, and vice versa for its memory strength parameters. This clear result is partly due to the model adaptation to the current task, which reassures clean strategy estimates and interpretations in each task, in line with our strong optimal-strategy instructions. Details can be found in the STAR Methods and supplemental information. Importantly, in RB, trial-wise strength in feedback-dependent stimulus processing for rule and modulation learning depended on the salience of the current gain ρ^I^ (difference between reward and no-reward trials), which was reliably different from zero, *M* = 0.87, *SD* = 0.16, *d* = 0.83, 95HDI[0.48, 1.18], BF > 1000. We further assumed that the salience of the accumulated rewards ρ^A^ moderates this effect over training (influence of ρ^I^ becoming smaller with accumulated reward), as well as reliably differing from zero *M* = 0.08, *SD* = 0.01, *d* = 1.14, 95HDI[0.75, 1.53], BF > 1000. In U, we measured between-stimulus differences in memory-updates via λ^*R*^ (gain for chosen stimulus relative to unchosen in reward trials) and λ^*NR*^ (gain for unchosen stimulus relative to chosen in no-reward trials), which both were reliably larger than zero, respectively, *M* = 1.45, *SD* = 0.12, *d* = 1.88, 95HDI[1.39, 2.38], BF_10_ > 1000 (chosen advantage with reward feedback), and *M* = 0.66, *SD* = 0.22, *d* = 0.45, 95HDI[0.14, 0.76], BF_10_ > 11.1 (unchosen advantage with no-reward feedback). However, the former advantage was stronger than the latter in direct comparison *M*_Diff_ = 0.72, *SD* = 0.26, *d* = 0.42, 95HDI[0.11, 0.73], BF_10_ = 7.3.

These aspects are especially important, since they informed our outcome-processing estimates in the fMRI analyses later in discussion. For better interpretation, and by means of cross-validation, we translated these estimates to corresponding trial-wise strengths shown in Figures 3E and 3F, with respect to chosen versus unchosen stimuli and reward versus no reward feedback, are explained in the STAR Methods and supplemental information (using ρ^A^ and ρ^I^ in RB, and λ^*R*^ and λ^*N*^*R* in U). Note, the pre-solution differences reflect strength estimates, while the interaction between pre- and post-solution periods reflects CAL’s hypothesis regarding an error-discounting mechanism, which treats every decision as correct as soon as CAL makes three consecutive confident and correct choices, predicting a drop in attention to the unchosen stimulus thereafter. Indeed, the estimates parallel the conclusions from the stimulus-attention data (Figures 3A and 3B), where we observed a processing advantage of the chosen versus unchosen stimulus in reward trials, and a switch in no-reward trials between the processing of both stimuli before the task was solved to an almost exclusive focus on the chosen stimulus after the task was solved. Notably, the strength estimates are achieved on qualitatively different parameters in RB (rule modulation) and U (memorization strength), but the error-discounting mechanism responsible for the pre-post solution interaction is shared between both modules.

Figures 3G and 3F also show CAL’s feature-attention estimates (i.e., averaging its rule-feature attention [α_*m*_] and modulator-feature attention [β_*m*_] on relevant and irrelevant dimensions, as done for the participants’ eye-movement data. Again, CAL readily predicts the observed trends, thereby replicating previous modeling results,^11^ concerning eye-tracking data on the deterministic single-stimulus versions of these problems.^5^ Most importantly, CAL predicts that feature attention in RB settles on both relevant dimensions only after participants solved the task, assuming that this reflects learned diagnosticity of – or the trust put into – its Simple Rule and its corresponding modulator, meaning that those representations reliably predicted different outcomes (subjective confidence). Overall, the match between CAL’s processing indices and the eye-tracking data on stimulus and feature attention lends strong credibility to our modeling hypotheses in both RB and U.

### Contrasting the mean brain activity between the two tasks

**Figure 4:**
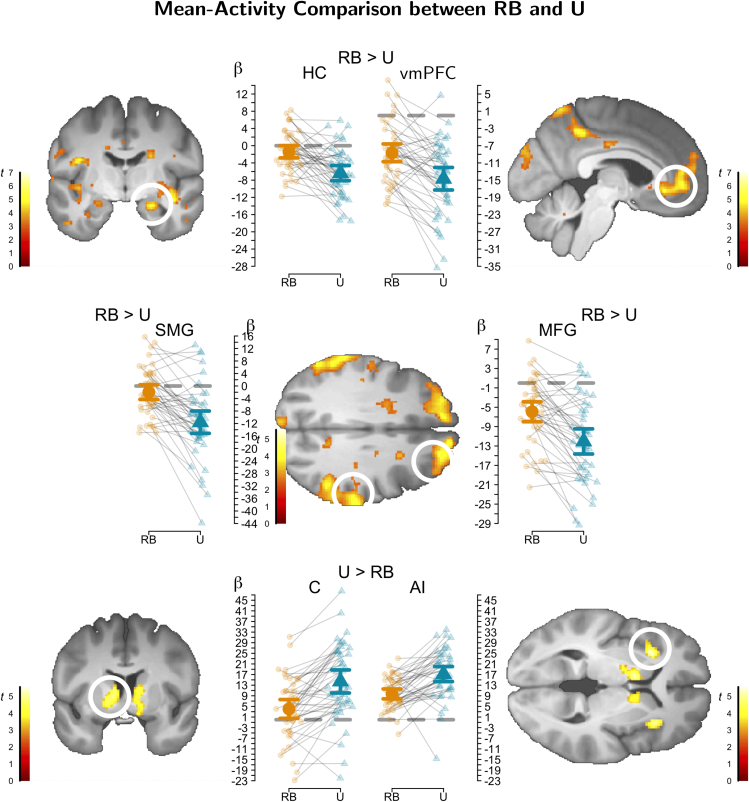
Contrasting mean BOLD activity between the two tasks Rule based [RB] versus unstructured [U] comparison. A) Greater activity in RB than U. B) Greater activity in U than RB. First level parameter estimates were corrected using VasA to account for inter-individual vascular differences^59^). Visualization threshold *p* < 0.001. Abbreviations: HC - hippocampus, vmPFC - ventromedial prefrontal cortex, SMG - supramarginal gyrus, MFG - middle frontal gyrus, C caudate. AI anterior insula, gray dashed line reflects *β* = 0. Error bars represent 95% confidence intervals.

In a first fMRI analysis that contrasted mean activity across all trials between the two tasks, we identified differential BOLD activity during choice in RB versus U (Figure 4; Table S1), which is meaningful because the average accuracy in both tasks was similar. We observed greater activity in RB in areas including the rostral inferior frontal gyrus, as well as areas of the frontoparietal control network, including the middle frontal, superior frontal, supramarginal, angular, and middle temporal gyri. In contrast, central nodes of the salience network showed greater activity in U than RB, including the anterior insulae, substantia nigra/ventral tegmental area (SN/VTA), and caudate. In addition, central nodes of the default mode network were more deactivated in U than RB, including the anterior hippocampus, vmPFC, and precuneus.

In a second analysis, we tested where the mean activity differed during feedback processing between both tasks. Interestingly, during the feedback phase, differences between both tasks were less pronounced, and we observed only greater activity in RB than U, in particular in the triangular inferior frontal and supramarginal gyri (Table S1).

### Distinct and overlapping brain areas in category abstraction learning-informed trial-wise analyses

**Figure 5:**
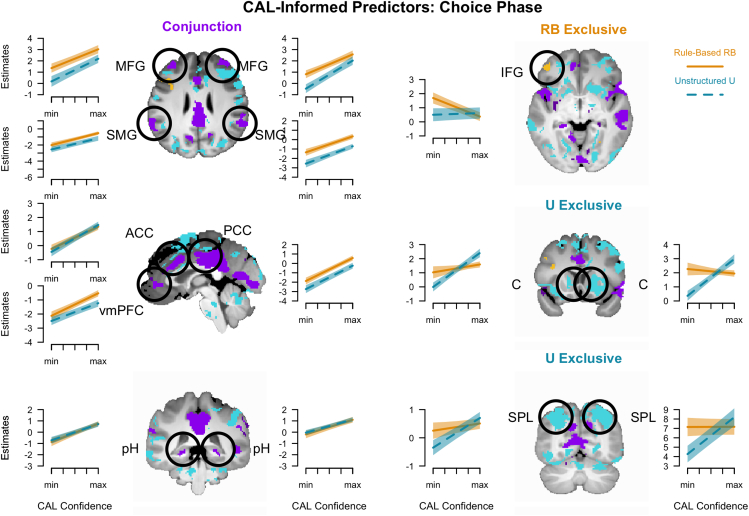
CAL-informed fMRI results of the choice phase (complete list of brain regions in Table S2) Highlighted areas correlated trial-wise with CAL choice confidence, either in both (conjunction, purple), exclusively in RB (orange) or in U (turquoise). Line plots: Marginal model estimates with ±1*SE*. The significant clusters are superimposed on the mean structural image of the participants. MFG - middle frontal gyrus, SMG - supramarginal gyrus, ACC - anterior dingulate cortex, PCC - posterior cingulate cortex, vmPFC - ventro-medial prefrontal cortex, pH - posterior hippocampus, SPL - superior parietal lobule, including, e.g., the intraparietal sulcus.

In the CAL-informed fMRI analyses, we used CAL’s individual trial-wise estimates and correlated them with the participants’ trial-wise brain activity separately for the choice and outcome phase. To identify strategy-unspecific areas, we conducted conjunction analyses, showing areas that correlated with the model-derived parameters in both RB and U. To identify areas specifically involved in only one of the two category learning problems, we applied exclusive masking (see STAR Methods). For the choice phase, we used each individual’s rule confidence in RB (Table 1, feature attention; Figure 3G, relevant dimensions) and memory confidence in U (reward probability of the chosen stimulus; Figure 2G). The two choice-confidence regressors mirror the learning trajectories, reflecting an increase in rule confidence when solving the task in RB and a rather continuous incremental increase in predictive strengths of memory associations in U. The conjunction analysis of rule and memory confidence revealed positive correlations with activity in - among other areas - the vmPFC, ACC and PCC (areas implicated in value based decision making), and the middle frontal and supramarginal gyri (areas of the frontoparietal control network), and the posterior hippocampi and precuneus (memory-related areas; Figure 5; Table S2). Unique negative correlations were observed with Rule Confidence in RB in the triangular IFG, the MFG, and the fusiform gyrus. No unique positive correlations were observed. In contrast, in U, all correlations were positive, with unique ones, among others, including the caudate and superior parietal lobule, thus suggesting a contribution to stimulus-based reward predictions.

**Table:** CAL parameters

Strength parameters	Cognitive functions
**Rule Learning Module**	

γ– Similarity/Contrast	Larger values … wider gradients → weaker rule learning
ω– Contextual Modulation	Larger values … stronger modulation/feedback sensitivity
ρ^*I*^– Reward Salience	Larger values … higher salience of current gain
ρ^*A*^– Reward Salience	Larger values … higher salience of accumulated gain

**Memory Module**	

λ– Configural Memory	Larger values … stronger memory encoding
λ^*R*^	Larger values … increased encoding of chosen stimulus
	after reward feedback, and less for unchosen stimulus
λ^*NR*^	Larger values … increased encoding of unchosen stimulus
	after no-reward feedback, and less for chosen stimulus

Derived Variables (fMRI)	Processing Relevance

**Rule Learning Module**	

Choice: *p*_*t*_(*A*)– Rule Confidence	Derived from α_*m*_ and β_*m*_ for truly relevant dimensions:
	Larger values … higher attention to/confidence in reward rule
Outcome: *v*_*t*_– Feedback processing	Derived from CAL’s obtained rewards, ρ^*I*^ and ρ^*A*^:
	Larger values … stronger extrapolation γ_*t*_ and weaker modulation ω_*t*_ in trial *t*

**Memory Module**	

Choice: *p*_*t*_(*C*)– Memory Confidence	Derived from memory-based predictions for chosen stimulus:
	Larger values … higher confidence/reward probability
Outcome: λ_*t*_– Feedback processing	Derived from λ, λ^*R*^, and λ^*NR*^, chosen stimulus processing:
	Larger values … stronger encoding after reward
	Lower values … weaker encoding after no-reward

**Figure 6:**
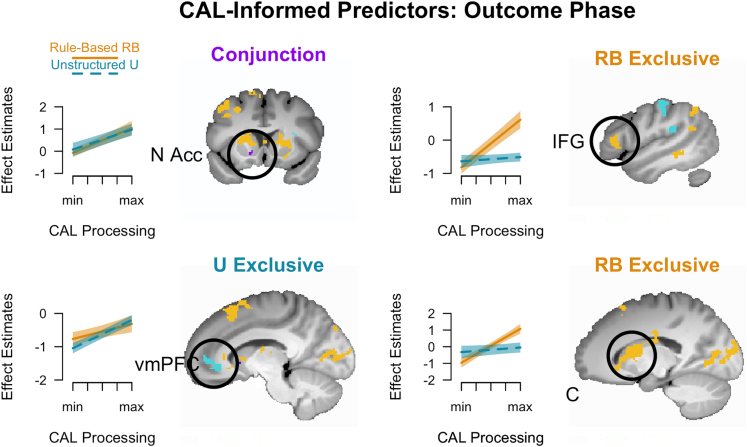
CAL-informed fMRI results during outcome phase Highlighted areas correlated trial-wise with CAL feedback processing, either in both (conjunction, purple), exclusively in RB (orange), or in U (turquoise). Line plots: Marginal model estimates with ±1*SE*. The significant clusters are superimposed on the mean structural image of the participants. N Acc - Nucleus accumbens, IFG - inferior frontal gyrus, and vmPFC - ventromedial prefrontal cortex.

For the outcome phase, we used the reward-dependent encoding strengths corresponding to the estimates shown in Figures 3E and 3F (see STAR Methods). We coded the latter with reference to the chosen stimulus. In U, this means higher values reflect positive reward signals, while negative values reflect error or no-reward signals (see also Figures S6 and S7). In RB, higher values reflect stronger rule updates in reward trials, but weaker modulation (and vice versa in no-reward trials). Thus, we considered positive and negative correlations accordingly. After learning, CAL predicts that the difference between reward and no-reward disappears due to feedback re-evaluation. The conjunction analysis of a positive correlation with activity in RB and U revealed only the N. accumbens as an overlapping outcome-processing area (Figure 6; Table S3). While there were hippocampal regions correlating with feedback processing in both tasks, these did not overlap. Most prominently, the triangular IFG as well as the caudate showed exclusive positive correlations in RB, whereas activity in the vmPFC correlated specifically in U. Only in U, we observed also negative correlations in some areas, including the middle frontal and angular gyri.

## Discussion

In this study, we characterize shared and dissociable cognitive and brain systems underlying rule abstraction and memorization during probabilistic value-based decision-making. To ensure sound inferences, we employed a within-participant design and joint cognitive modeling of behavior and eye movements to inform analyses of brain activity in two learning tasks, representing RB and U problems.^7^ In those, participants predicted in repeated trials which of the two presented stimuli would yield a reward. Here, we discuss the main implications regarding theories of learning and their neural correlates, based on the behavioral and fMRI results.

The behavioral analyses of the learning trajectories strongly supported the idea that rule-learning is characterized by a sudden rule discovery in RB, while memorization is characterized by rather incremental learning in U (Figure 2). As rule-discovery comes with the notion of selective feature processing, accordingly, RTs quickly reduced in RB relative to U after solving the task, mainly due to reduced attention to the irrelevant dimension in RB. CAL’s dual module architecture^11^^,^^60^ successfully accommodates both the distinct learning trajectories and the changes in feature attention (Figure 3), but also shared aspects, which help elucidate the cognitive processes underlying learning from abstraction on a broader scale.

Specifically, in RB, focusing of feature attention occurred only after rule discovery, which has been previously interpreted as an argument against theories assuming explicit hypothesis generation and testing during the search phase,^61^ as this would entail attending only to tested features (i.e., try two dimensions, and if incorrect, two other).^5^^,^^6^ In contrast, the current results rather support the idea that attention follows, and not precedes, the discovery of rule solutions.^62^ CAL assumes that rule discovery is driven by first building up Simple Rules (e.g., square antenna predicts reward and diamond antenna predicts no reward), and then by recognizing under which conditionals (modulators) this rule can be applied or not (apply if one-circled wing, reverse if two-circled wing). CAL then focuses attention on the corresponding feature dimensions. This idea implies that feature attention is success-driven rather than updated due to behavioral errors during the search phase, known as error-driven attention learning.^4^ Otherwise, the re-focusing of feature attention to only the relevant dimensions should have occurred prior to solving the task. In contrast, in U, attention to all three dimensions throughout the entire experiment implicates configural processing of all features as assumed in CAL’s Hebbian learning, resonating with the incremental increase of associative strength during memorization.

Importantly, we also found similar stimulus-attention patterns (fixation of chosen vs. unchosen stimulus) in RB and U. During the choice phase, participants more strongly attended to the chosen compared to the unchosen stimulus in both RB and U, indicating reward anticipation, and/or response preparation (a.k.a., gaze-cascade effect).^57^^,^^58^ During outcome processing, prior to solving the RB or U tasks, obtaining reward invigorated attention paid to the chosen stimulus, whereas receiving no reward led to nearly equal allocation of attention to both stimuli. However, this equal distribution of attention changed after solving both tasks, as participants preferred to attend the chosen stimulus and ignored the unchosen one, even in no-reward trials. Normally, this expectancy violation would be assumed to trigger a reorientation of attention. However, likely due to fully knowing the predictive stimulus-outcome associations, this now uninformative feedback was ignored or re-evaluated. These changes in attention allocation after solving the tasks are consistent with CAL’s modeling hypotheses (and simulations, Figure 3), suggesting that after solving the task, no-reward feedback regarding the chosen option is re-evaluated as being rewarding. While there exist alternative error-discounting assumptions,^4^^,^^63^^,^^64^ CAL’s hypothesis that participants continued learning in a semi-self-supervised fashion (accepting reward as is, but re-evaluating errors) provides a viable path to explain behavior, attention, and neural patterns.

Taken together, CAL’s performance in accommodating the data lends strong support for its learning hypotheses. Intriguingly, while we assumed that the RB problem is solved via learning Simple Rules and their conditional modulators, and that the U problem is solved via incremental stimulus memorization, the current results imply that reward equally affects attention allocation, yet with different consequences. Receiving a reward, in CAL’s rule module (RB), enhances rule extrapolation, while errors enhance the learning of their conditionals. In CAL’s memory (U), however, the descriptive results suggest that reward more strongly increases stimulus-outcome associations than errors, that is, before eventually solving the task. After solving the task, CAL then protects its learned solutions in both modules by re-evaluating error feedback according to its own choices.

The analysis of our neural data revealed that these fundamentally different learning strategies recruit distinct brain networks. A comparison of the mean activations in both tasks revealed that the Frontoparietal Control Network is strongly involved in RB than in U, suggesting that rule abstraction and application involve more cognitive control that flexibly adapts behavior to the current context (Figure 3).^65^^,^^66^^,^^67^ In U, however, parts of the Salience Network, including the anterior insula and caudate, showed stronger activation, suggesting that memorization requires maintaining arousal to a larger degree, but which also could concern task sets and aspects of sustained control.^68^^,^^69^^,^^70^ Activity of the caudate during both U and RB has been previously implicated as cognitive control based on its role in cortico-striatal control loops.^23^^,^^28^^,^^71^^,^^72^ However, our data clearly shows that this brain region is more active in U than RB (note that it was also activated in RB in the current task), an inference made only possible by our within-subject design that directly compares brain activity during both tasks in the same participants. Finally, we observed a stronger deactivation of the default network during U compared to RB (Figure 3), consistent with its down-regulation caused by the salience network.^73^ Taken together, we observed two cognitive control networks, the Frontoparietal Control Network and the Salience Network, consistent with differences in task orientation.

Using CAL-informed analyses to interrogate the fMRI data during the choice phase, we identified common, but also unique brain networks specifically involved in rule abstraction and memorization (Figure 6). We individually correlated CAL’s rule confidence and memory confidence, trial-by-trial, with brain activations. Both predictors increase over time, indicating a heightened fidelity of the respective CAL module, but with different slopes: Rule confidence exhibits a quick increase following rule discovery, whereas memory confidence increases only gradually, and also before reaching high levels of accuracy.

These analyses unveiled a unique negative correlation of rule confidence with left triangular IFG activity in RB (but also with MFG and fusiform), which would be consistent with its previously suggested role in rule evaluation,^24^ as well as its suggested more general role in logical reasoning and transitioning between stimulus-rule mappings.^74^^,^^75^^,^^76^^,^^77^ Although peak-activity locations in MFG and triangular IFG vary between studies, both areas are implicated in learning modulatory structure, as distinct from associative stimulus-response learning.^17^ The Type II problem reflects such a modulatory structure, solved by modulating a single-dimensional rule via context features (rule execution vs. inhibition/reversal/remapping), which is also the theoretical basis of our CAL model.

Memory Confidence in U exhibited exclusively positive correlations with the putamen/caudate (head and body) among other areas, conceptually replicating previous results on unstructured learning tasks.^25^^,^^27^^,^^28^^,^^29^ This conceptual replication is non-trivial and theoretically relevant because it supports the generality of the finding, given the substantial differences in the experimental design between the current and the cited studies with respect to stimuli and procedure. Note, however, that this activation lies anterior to the other caudate subregion, in which we found stronger mean activity in U compared to RB reflecting the involvement of cognitive control by the salience network (contrast of mean activity, Figure 4). The activity of this caudate subregion correlating with increasing memory confidence appears to be involved in the acquisition of the action-outcome contingencies or stimulus-response learning.^78^^,^^79^ That the adjacent caudate subregions might feature differential neurocognitive processing, cognitive control, and stimulus-response learning would be consistent with the diverse connectivity patterns of the caudate.^28^^,^^80^

The superior parietal lobe/intraparietal sulcus, which we also observed in the current task, has been implicated previously together with the caudate in unstructured learning and tracking of task specific exemplar information and exemplar based similarity.^81^^,^^82^^,^^83^ This region also represents memory specificity for items and features, which is necessary for exemplar-based choice.^84^

Despite the sudden (RB) versus incremental (U) increase of the confidence estimates, we also found common effects for both tasks in memory, reward anticipation, and control networks. A hippocampal and precuneal contribution to memorization was expected based on its role in recollection-based declarative memory for unstructured, yet verbalizable stimulus-categories.^2^^,^^82^ However, our finding of additional hippocampal involvement in RB suggests that this brain region could also be engaged in the type of relational reasoning necessary for rule discovery.^22^^,^^85^ Overall, this finding highlights that declarative mnemonic processing facilitates both rule abstraction and idiosyncratic memorization. In this vein, previous categorization studies associated variations in posterior hippocampal activity with recognition strength for simple, 1-dimensional rules but also exemplar- and prototype-based processing.^81^^,^^86^^,^^87^ It should be noted, however, that in two of these studies, anterior hippocampal activity was also associated with exemplar-based processing.^81^^,^^86^ Intriguingly, however, research further suggests a role of the anterior hippocampus in the encoding/updating of attention-compressed stimulus representations.^26^^,^^86^^,^^88^In other words, these and our results would be generally in line with the idea that the hippocampus codes stimulus-stimulus (S-S) bindings, which have been differentiated from stimulus-response (S-R) bindings in previous studies.^31^ Disentangling the full details of these dependencies is beyond the scope of this research article and warrants a thorough treatment in a review or meta-analysis.

Another common network (vmPFC, ACC, and PCC) has often been associated with the processing of subjective value and reward anticipation.^22^^,^^89^^,^^90^^,^^91^ Consistently, our observed increase in activity in these regions to Rule and Memory Confidence parallels these earlier findings, because higher confidence implies a greater expectation and anticipation of reward. In particular, the vmPFC has been implicated in a variety of learning-related cognitive functions such as the monitoring of the reliability of predictive models.^92^ In the context of category learning, several reports point to a role in learning to abstract categorical information from individual stimuli, such as representations of prototypes^86^ and the transfer of complex rules.^82^ However, this region has also been involved in exemplar-based category learning,^84^ which exhibits some resemblance to our unstructured, memory-based learning task. Consistently, we find stronger vmPFC correlations with CAL’s feedback processing variables in U compared to RB, reflecting processes of error monitoring and discounting. Finally, category learning has a common conceptual basis with schema learning^93^ and retrieval of schema information also recruited the vmPFC,^94^ which might parallel its involvement in the retrieval of category information during choice, as in the current study.

In addition, activity in areas involved in control processes (MFG, SMG, and middle temporal gyri [MTG])^70^ also correlated with Rule and Memory Confidence in both tasks. As mentioned above, mean activity in a subset of these areas (Figure 3) was greater for RB than U, suggesting higher relevance of flexible control processes for rule abstraction.^65^ Intriguingly, common activity in reward anticipation and control networks is consistent with the preferential neurocognitive processing of the chosen stimulus, resembling the similar evolution of stimulus attention across learning in both tasks (gaze-cascade effect) as measured in the fixation patterns. Rule and memory confidence, expected to increase throughout learning, positively correlated with activity in these areas, which implies greater involvement of the respective neurocognitive processes (memory, reward anticipation, and flexible cognitive control) after solving both tasks. Consistent with this notion, precuneal activity has been specifically associated with retrieval, which comes into play mostly after learning, but not encoding.^95^ Interestingly, our data suggest that flexible cognitive control is also more involved after learning than before, in line with the idea in CAL that its cognitive control increases by accumulating reward. However, as mentioned, we observed a negative correlation, that is, more involvement before than after learning, only for RB in the IFG (and MFG and fusiform gyrus), consistent with its active role in rule evaluation.

In summary, the roles of these shared networks of areas identified by CAL-informed choice-confidence regressors are not restricted to rule-based and unstructured probabilistic category learning but are more generally active in tasks such as value-based decision making with similar cognitive demands with respect to, for instance, cognitive control, feedback processing, declarative or working memory. Furthermore, because our task did not include a low-level control condition, we are not able to disentangle the unique contributions of each brain region in the network. Determining which of these processes is central to the shared activity warrants further research.

The CAL-informed analyses of the outcome phase, that is, the trial-by-trial correlation between brain activity and task-specific variables of feedback processing, also revealed unique and common brain regions in both tasks. For RB, this analysis provided strong support for rule-relevant outcome processing in the caudate (head, body, tail) and triangular IFG (more posterior relative to choice). These positive correlations indicate higher activity in reward than no-reward trials before rule discovery and declining activity once solving the task, which seems in line with some previous research.^72^ This is expected for processes involving rule evaluation when confronted with feedback,^24^^,^^96^^,^^97^^,^^98^ which would be further consistent with the update of theory representation in theory-based learning.^17^^,^^77^ According to CAL’s learning hypotheses, the RB results suggest that category rules become more strongly established – through simple rule extrapolation – when participants receive rewards compared to when they do not, while in error trials, more complex structural adjustments are enhanced. This may also relate to how participants interpret errors in probabilistic tasks before they know the correct solution; for example, errors may only be confidently used to guide learning if they are perceived as reliable teaching signals. Other studies interpret these regions as part of cortico-striatal loops supporting executive control and motivation.^27^ Moreover, activity in hippocampal clusters correlated exclusively with outcome processing in RB (Table S3), potentially due to the declarative encoding of updated rule features as highlighted above.

Activity in the vmPFC and different hippocampal clusters showed a positive correlation with outcome processing in U. This might imply the reward-driven encoding of stimulus-outcome associations in a Hebbian fashion and/or the assimilation of those into the existing knowledge networks.^94^^,^^99^ It may also reflect the formation of stimulus-stimulus associations.^31^ In contrast, negative correlations during feedback processing in U were found in a few cognitive control regions (MTG, angular gyrus, anterior insula), indicating stronger responses to no-reward than reward trials before the correct solution was known. From a CAL perspective, this suggests responses to errors (no-reward feedback) that trigger the model’s search for modulatory contexts – stronger after errors, but reduced once the solution is learned due to error discounting. Overall, the results support both an error-signal interpretation and enhanced context-dependent structuring of simple feature-outcome rules. Finally, there was only one region common in both tasks, the N. accumbens, consistent with its well-established role in reward (but also corrective feedback) processing.^25^^,^^30^

### Limitations of the study

While there are clear parallels, it is an open challenge to fully align our findings with previous studies. This is complicated due to differences in theoretical frameworks and methodological choices. Here, we mainly addressed the cognitive processes of modulatory rule learning,^15^^,^^17^ contrasted from those assumed to reflect configural/idiosyncratic exemplar memory,^3^ but our tasks have been approached differently in previous studies. Mack et al.^88^ found stronger vmPFC involvement in RB than U tasks using a clustering model reflecting the strength of feature-focusing or entropy, similar to our RB choice confidence, whereas we observed no such task differences in our study. This likely reflects variations in experimental design, such as the use of pre-training and instructions. Those methods apparently prevented the classical finding of substantial performance differences between tasks/strategies in our study.^7^^,^^92^^,^^100^ Similarly, Bowman and Zeithamova^86^ reported IFG involvement in exemplar learners, which contrasts with our Type II findings. However, this may reflect a more meta-theoretical aspect that exemplar-based accounts are plausible to address rule-plus-exception learning, as in their study, but modulatory-learning theory is equally suited, as indicated by the current CAL findings in the IFG, providing a viable theoretical alternative to learning in non-linear category structures.^11^ To align insights across studies, we therefore suggest a process distinction between simple-rule learning, context-dependent rule modulation, and exemplar memory (e.g., enumeration of features).

In summary and conclusion, we used a recently developed, sophisticated computational model, and investigated two fundamentally different, yet frequently used learning strategies - rule abstraction and memorization - and comprehensively characterized their distinct behavioral, physiological, and neural profile. A defining feature of rule abstraction is the sudden increase in learning trajectories after rule discovery, which corresponds to a sudden drop in reaction time due to a focusing of feature attention when the irrelevant stimulus dimensions are ignored. Memorization, on the other hand, is characterized by a gradual increase in learning trajectories and an equally gradual decrease in response times due to the sustained attention to all features throughout the experiment, as no dimension can be ruled out. The recently developed computational model (category abstraction learning, CAL) predicts these trends by its architecture, combining modulatory learning and exemplar memory, ideally suited to capture the cognitive processes that these learning strategies engage. Our CAL-informed analyses of neural activity revealed areas uniquely supporting both learning strategies, namely rule evaluation and stimulus-outcome association, consistent with the differential feature attention, as well as common networks involved in reward anticipation and cognitive control, consistent with the flexible allocation of stimulus attention. Thus, comparative analyses of reinforcement learning-based neural models^22^^,^^41^ and the class of state-transition or modulatory learning models,^17^^,^^101^ including CAL,^11^^,^^42^ hold the promise for deepening our understanding of the mechanisms that drive various strategies in decisions under uncertainty.

The broader implications of our findings for understanding the neural mechanisms of learning are 2-fold. First, the observed interaction between task-specific regions (e.g., those processing rules or categories) and more domain-general areas (such as those involved in cognitive control or memory integration) suggests that this kind of coordinated brain activity is not unique to category or value learning. Instead, it likely reflects a more general principle of how the brain supports learning across a wide range of contexts, including those that involve uncertainty, abstract reasoning, or flexible rule application. Second, our results provide evidence that learning stimulus-reward associations within rule-based frameworks can be effectively explained by modulatory learning mechanisms – such as those formalized in the CAL model – rather than relying solely on traditional prediction-error-based learning models. This suggests that learners may adapt their behavior not just through error correction, but also by actively searching for and integrating higher-order contextual cues that shape how simple stimulus-reward relationships are interpreted and used.

## Resource availability

### Lead contact

Requests for further information and resources should be directed to and will be fulfilled by René Schlegelmilch (r.schlegelmilch@uni-bremen.de).

### Materials availability

This study did not generate new materials.

### Data and code availability

• The supplementary material, model, data, and scripts for the behavioral and eye-tracking analyses can be found on https://osf.io/f6azc/.
• For data and scripts regarding the neuroimaging analyses, please contact Tobias Sommer (t.sommer@uke.de).

## Acknowledgments

This work was supported by the Landesforschungsförderung Hamburg (LFF FV38). JG was also supported by the Collaborative Research Center 1528 “Cognition of Interaction” and the Research Unit 5389 “Dynamic Belief Updating” funded by the 10.13039/501100001659German Research Foundation. We thank Kira Diermann for help with data acquisition.

## Author contributions

A.D., G.J., J.G., and T.S. conceptualized and designed the study. A.D. finalized and carried out the study being part of her doctoral thesis. R.S. analyzed the behavioral and eye-tracking data, designed the computational model, and conducted the modeling analyses. R.S. and T.S. drafted the article. T.S. analyzed the fMRI data. R.S., G.J., J.G., and T.S. provided critical revisions of the article.

## Declaration of interests

The authors declare no competing interests. Alina Dinu is an employee of OTTO GmbH & Co. KG, Werner-Otto-Straße 1–7, 22179 Hamburg.

## STAR★Methods

### Key resources table

**Table:** 

REAGENT or RESOURCE	SOURCE	IDENTIFIER
**Software and algorithms**		

Psychophysics Toolbox Version 3 (PTB-3)	https://github.com/Psychtoolbox-3/Psychtoolbox-3	
Matlab R2014b	https://www.mathworks.com/products/matlab.html	
R jags	https://cran.r-project.org/web/packages/rjags/index.html	
afex	https://cran.r-project.org/web/packages/afex/index.html	
emmeans	https://cran.r-project.org/web/packages/emmeans/index.html	
DEoptim	https://cran.r-project.org/web/packages/DEoptim/index.html	
SPM12	https://www.fil.ion.ucl.ac.uk/spm/software/spm12/	
Category Abstraction Learning (CAL) model	https://osf.io/f6azc/	

**Other**		

fMRI Scanner		Siemens Trio, 3T
EyeLink Eye-Tracking system		EyeLink 1000, SR Research
MR compatible screen		NordicNeuroLab, resolution: 3840 x 2160, pixel pitch 0.076225 (H) x 0.2247 (V), refresh rate 60 Hz
MR compatible button box		

### Experimental model and study participant details

The study was approved by the local Ethics Committee of the Hamburg Medical Association (PV5947). The experimental task was implemented using Psychophysics Toolbox Version 3 (PTB-3) running in Matlab R2014b (Mathworks, Natick, MA, USA). Participants were screened for history of psychiatric or neurological disorders and current use of psychoactive medications. For eye-tracking reasons, participants with diopters below -4 or above +4 were not invited to take part in the study, which was run in two sessions on separate days. 9 of the 53 invited participants could not be analyzed either due to technical failures or because they showed up only once (remaining sample *N*=44, 29 females, 25.5 ± 3.1 years old, range 20 -32 years). Participants were compensated with 30 Euros for participating in Day 1 and 25 Euros for participating in Day 2. Depending on their performance, they could earn up to 5 Euros more on each testing day, in particular the outcome of 10 randomly selected trials were summed up after finishing the task and served as additional bonus.

### Method details

#### General procedure

Each participant solved the two problems on one of the two testing days (order randomized between; 16.7 ± 21.9 days apart, range 2 – 112 days). Each problem (RB versus U) was performed with a different stimulus set. In the final sample 25 participants solved the rule-based task with the ‘butterflies’ stimulus set, 21 participants did the RB task first.

On each testing day, participants were explicitly instructed whether the task should be solved with a disjunctive rule (RB) or based on memorization of the individual stimuli (U). Then they performed a pre-training task for the respective problem outside of the scanner. The pre-training was identical to the main task, except using different stimuli (Figure S2), the feedback was deterministic, participants were told which dimension was irrelevant in RB, and they only trained until reaching a learning criterion (15 consecutive correct trials; see supplemental information). After this, the concept of 80% probabilistic feedback was explained and they continued with the same task including probabilistic feedback. Then participants entered the MR scanner and started with the experiment.

In the MR scanner, participants first completed an ‘Exposure task’ in which the 8 stimuli of the main experiment were presented individually on the screen, each 4 times. The Exposure task was introduced for multivariate analyses (not reported). Then they performed 5 runs with 32 trials each of the main learning task followed and finally another run of the exposure task. The stimulus presentation screen with a resolution of 1920 x 1080 pixels was mirrored into the scanner using an MR compatible screen from the NordicNeuroLab (resolution: 3840 x 2160, pixel pitch 0.076225 (H) x 0.2247 (V), refresh rate 60 Hz). Responses with the index and middle finger of the dominant hand were recorded using an MR compatible button box.

#### Stimuli

Two sets of eight stimuli were created for the two main tasks, either depicting robot-like figures (humanoids, Figure S1) or butterflies (Figure 1), as a compromise for tracking of fixation on the individual features and while still perceivable as holistic objects. The size of the stimuli was 3.77 x 11.97 degrees of visual angle. In both sets, the stimuli differed in three features: color, number and orientation, each with binary dimensions. In both stimulus sets, the varying dimensions were equidistant from each other and were of the same size, approx. 0.83 x 0.83 degrees of visual angle.

#### Main learning task

After being familiarized with the stimuli to avoid novelty effects (exposure task), a categorization trial started with the choice phase presenting two stimuli, one of each reward category (Figure 1). Participants had 4 seconds to choose the preferred stimulus using the left or right button of a response box. The chosen stimulus was surrounded by a grey rectangle until the end of the choice phase. A black fixation cross on a white background followed for 2 or 4 seconds (randomized across the task). In the following feedback phase, the two stimuli were presented for 2.5 s where the chosen was highlighted. The outcome was indicated by a 50 cents coin/a 50 cents coin crossed by a red X and the updated cumulative score at the bottom of the screen. If the participants failed to respond within the time frame, the feedback screen was blank and only displayed the sentence “Please respond faster!”. The trial ended with a fixation cross presented for 3 seconds (we discarded these trials in the eye-tracking and fMRI analyses). Irrespective of their performance, participants had to complete 160 trials that were distributed in 5 runs with 32 trials each.

The stimulus presentation ensured that two stimuli in each trial always differed on at least one feature, limiting the number of possible pairings to 16, which were pseudorandomly presented 10 times with a minimum of one trial distance before the same pair was presented again. The 20% misleading probabilistic feedback was pseudorandomized appearing for 2 out of the 10 repetitions for each pair.

#### Eye-tracking

Eye movements were recorded under constant lighting conditions from the right eye using the Eyelink Eye-Tracking system (EyeLink 1000, SR Research, Ottawa, ON, Canada), with a sampling rate of 500 Hz and a spatial resolution of 0.01 and a spatial accuracy of 0.5. The screen resolution was 1920x1080px. Stimuli were presented equidistant from the screen center, located at the middle of the corresponding side. Each feature was about 3 cm^2^, translating to a visual area of 0.83 x 0.83 degrees (1.73 cm x 1.73cm). The eye-tracker was re-calibrated before every 32 categorization trials (5 runs).

For both left and right stimuli, three equally sized areas of interest (AOIs) were defined, each containing one of the three features. The AOIs were assigned to the fixations in three steps, as there was some drift error due to the mirror attached to the apparatus in the scanner. In step 1, we calculated the drift as deviation from the center of the screen for each individual, across all trials within each run separately. We then shifted all fixation coordinates in that run using the mean x and y coordinates of the fixations during the fixation cross phases serving as baseline. In step 2, we determined, across all participants and trials, the spatial coordinates at which the number of fixations were most frequent in the choice phase, reflecting the data-driven center of each stimulus feature, which were 750, 525, and 300 for the y-axis, and 576 and 1344 for the x-axis. In step 3, we drew non-overlapping AOIs with 110px plus/minus the derived center of the AOI.

To ensure data quality, we removed all runs of participants after visual inspection, in which the fixation drift included a radial tilt of the fixations thereby largely falling outside the top and bottom AOIs, and thus could not be corrected by vertical or horizontal shifts. We also removed runs with overall fewer fixations than trials. This led to a total of 53 excluded runs, 24 and 29 in RB and U, respectively. The remaining sample consisted of 39 participants with data in at least one run in one of both tasks. Specifically, in RB, there were 30 participants with complete data for all runs, 4 participants with 4 complete runs, otherwise either 1, 2, or 3 runs. In U, there were 31 participants with complete data for all runs, 5 participants with 4 complete runs, otherwise either 2 or 3 runs.

#### Humanoid stimulus set - Main task

As highlighted before, all participants solved both RB and U problems on separate day sessions. In each problem, we used an own stimulus set, randomized between participants. The butterfly stimulus set is reported in Figure 1. Figure S1 shows the second stimulus set (humanoids).

#### Pre-training practice task

As for the main task, we used two separate sets of eight stimuli for the pre-training task. Each set consisted of geometrical figures with three dimensions. The first set contained figures, which varied in color (red or blue), shape (square or triangle) and filling (filled or not filled). The second stimulus set contained figures which also varied in color (green or purple) and shape (star or pentagon) but had contour as third dimension (with or without contour), illustrated in Figure S2.

For the deterministic RB practice task one of the two sets of stimuli was randomly chosen. The eight stimuli were shown to the participants before starting the practice task. As in the main task, category assignments were done based the category structures and were told to identify the valuable stimuli. For the disjunctive rule, they were told only in the practice RB task which feature was irrelevant (the filling or the contour dimension). The trial timing was nearly identical to that in the main task, except showing both stimuli for 3.5 s (instead of 4s) during the choice phase. After an interval of 1 -3 s feedback was presented and the next trial followed after a 1 second inter-trial interval (instead of 3s). The task stopped after 15 consecutive correct trials. At the end of the task, the participants had to report the underlying rule (and all participants successfully did).

The probabilistic part of the practice task started with a text explaining the concept of probabilistic feedback using the example of an 80% reward contingency. The participants then continued with the practice task, but instead of deterministic feedback they received probabilistic feedback. Thus, participants knew which stimuli formed the valuable category, and the procedure aimed to familiarized them with the probabilistic feedback. After approximately five minutes, the task stopped. Only the deterministic practice task on the first testing day was followed by its probabilistic version because the concept of probabilistic feedback was identical fro RB and U.

#### fMRI data acquisition and preprocessing

Functional magnetic resonance imaging (fMRI) was performed on a 3 T system (Siemens Trio) with a 64-channel head coil using an echo planar imaging T2∗-weighted sequence with 54 contiguous axial slices (TR 1.63 s; TE 29 ms; flip angle 70°; 2-mm thickness with 0.5-mm gap; field of view 224 × 224; multi-band mode, number of bands: 2, interleaved phase encoding in descending order; PAT factor 2). For spatial normalization, a whole brain T1-weighted structural MR image was acquired using a 3D MPRAGE sequence (TR 2300 ms, TE 2.89 ms, flip angle 9°, 1-mm slices, field of view 256 × 192; 240 slices).

Functional imaging data were preprocessed using Statistical Parametric Mapping 12 (SPM12, Wellcome Department of Cognitive Neurology, London, UK). To prevent biases due to spin saturation, the first five functional images were discarded. To correct for movement and susceptibility-by-movement artifacts, all functional images were realigned and unwarped. Individual structural T1 images were then coregistered to the functional images. Coregistered T1 images were segmented into gray and white matter, which were subsequently used within the ‘diffeomorphic anatomic registration through an exponentiated lie algebra algorithm’ (DARTEL) toolbox to create individual flow fields. Those were used for normalizing structural and functional images to MNI space. The functional images were sampled with a voxel size of 2 x 2 x 2 mm^3^ after normalization. Finally, functional images were smoothed with a full-width half maximum Gaussian kernel of 6 mm in all spatial directions.

### Quantification and statistical analysis

#### Bayesian learning accuracy analyses

For analyzing learning accuracy, we employed a hierarchical Bayesian logistic regression using R jags.^102^ To accommodate the sudden solution trends in RB and to estimate the moment of solving the task (*t*_*s*_), we modified the logistic regression in the following way. As a hierarchical estimate of *t*_*s*_ we defined a relatively unconstrained population prior of θ∼Dirichlet(1,…,1) (with 160 indices, one for each trial; this moment seemed non-normally distributed). For each individual *s*, we then sampled a corresponding trial index *o*_*s*_∼Categorical(θ) (i.e., onset of learning). Before *o*_*s*_, accuracy predictions were fixed to ${\hat{p}}_{st}=.5$ in a given trial *t*, and afterwards applied a standard logistic function with, ${\hat{p}}_{st}=1/\left(1+\exp\left(-b_{s}\cdot \left(-o_{s}+t\right)\right)\right)$, where adding *t* to *o*_*s*_ means that the formula yields ${\hat{p}}_{st}=.5$ at *o*_*s*_ and increasing in a limited growth in subsequent trials. The parameter *b*_*s*_ reflects an individual’s estimated slope or strength of increase in accuracy, sampled hierarchically from *b*_*s*_∼Gaussian[0,3](β^*M*^,1/(β^*SD*^)), with β^*M*^∼Uniform(0, 3) and β^*SD*^∼Gaussian(.1, 1). Finally, the resulting predictions were applied via Bernoulli likelihood link, $p_{st}∼\text{Bernoulli}\left({\hat{p}}_{st}\right)$. This method was applied to each task separately. The individual estimates of *b*_*s*_ were based on the mean of the individual posterior distributions. We derived *t*_*s*_ using the posterior behavioral predictions from all iterations taking the trial in which the function predicted 80% accuracy first in most of the iterations (maximum likelihood).

#### General statistical analyses

In all following analyses we used mixed-effects regressions R package afex:mixed^55^^,^^103^; using Type III LRT tests for comparison (full model versus reduced model without the tested effect). We always included by-participant random intercepts. We included random slopes as long as they did not lead to singular model fits or parameter identification issues (e.g., in the eye-tracking analyses, due to missing observations). Regarding *t*_*s*_ analyses, we always coded the 160 trials into blocks of 32 trials each, such that *t*_*s*_ represented the last trial in block 0. Thus, blocks ¿ 0 represent post-solution trials (in all Figures block 0 = *t*_*s*_ on the x-axes). However, to cope with very quick learners or non-learners, we always coded at least the first ten and the last ten trials as ‘before’ and as ‘after’ the solution, respectively. We then aggregated the dependent variables within these blocks for each individual and task, and relevant design cells (e.g., by stimulus). Similarly, to prevent singularity estimates due to blocks hardly observed (e.g., block of -4), we collapsed all blocks =<–2 and blocks =>5.

#### Stimulus attention in outcome phase depending on reward

We tested whether dwell times (DTs) differed between the chosen and unchosen stimulus in the outcome phase, depending on whether participants received reward or not. We present further analyses in the Supplements concerning gaze-cascade effects during choice and outcome phases (Figures S3 and S4). We derived the corresponding DT by summing all DTs in a given trial during the outcome phase, with respect to both stimuli. In the mixed-effects model, we entered task, blocks, reward, and stimulus with all main effects and interactions. As random effects, we entered by-participant intercepts, and by-task and by-stimulus random slopes.

#### Feature attention in choice and outcome phase

Regarding feature attention, we also included a comparison between the choice and outcome phase fixations to test whether the irrelevant feature is ignored during both, which would speak for continued updating, rather than neglecting feedback altogether (e.g., in case of an interaction, feature attention during the outcome phase might be non-functional). We calculated the summed DTs on each feature in a given trial and phase, but regardless of left or right stimuli. We then averaged the DTs of the two relevant features in that trial in RB, and did the same for the spatially matched features in U for comparing the DTs between relevant versus irrelevant features and between both tasks. The mixed-effects model included blocks, task, phase, and feature as factors, with all main effects and interactions. As random effects, we assumed, by-participant random intercepts, and by-task and feature random slopes (we did not add by-block slopes due to missing observations).

#### fMRI analyses

Event-related BOLD responses were analyzed using general linear models (GLM) in a mass univariate approach as implemented in SPM. On the subject level, we concatenated the five runs of each task, but implemented run-specific AR(1)-autocorrelation for serial dependencies, high pass filter (128 s) for baseline drifts as well as constants in the GLM. The two tasks were treated as two sessions in SPM. The first-level parameter estimates were scaled by proxies of the local vascularization using VasA to account for inter-individual vascular differences.^59^

#### Contrasting mean activity between the two tasks

To identify differences in mean activity between RB and U across all trials, a first-level model included regressors for the choice and the outcome phases of the trials. These onset regressors were generated by convolving a delta function containing the onsets of the events with the canonical hemodynamic response function. In addition, we used 6 movement parameters as nuisance regressors. On the second level, the parameter-estimates of the onset regressors were contrasted in a 2 (choice versus outcome phase) x 2 (RB versus U) factorial linear model.

#### Category/value abstraction learning parameters

The Category Abstraction Learning (CAL) model is a set of category-learning hypotheses linked to context-dependent learning^104^^,^^105^ and occasion setting.^15^^,^^16^^,^^17^^,^^42^ CAL’s core mechanism–modulation of Simple Rules (see Figure 7) seeks to explain how humans flexibly acquire, apply, and adapt categorization rules in a variety of benchmark tasks with multi-attribute stimulus classification,^11^ especially enabling CAL to discover disjunctive (Type II) rule structures. In that tradition, CAL seeks to maximize the *probability* of reinforcement, and we accordingly treat Reward’ and No Reward’ as categorical labels/responses A′ and B′, rather than concerning stimulus value or magnitude.^30^^,^^92^^,^^106^^,^^107^^,^^108^^,^^109^^,^^110^^,^^111^^,^^112^ Importantly, we here extend CAL to handle probabilistic feedback in the stimulus-selection task, for CAL-informed strategy-specific process estimates.

**Figure:**
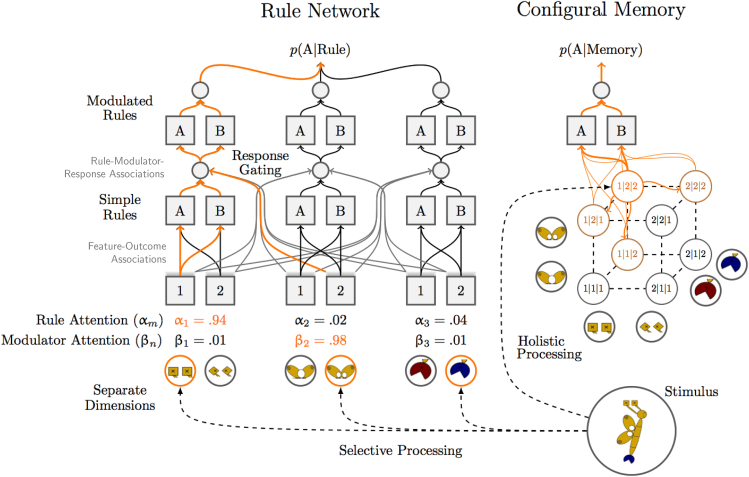
CAL schematic Rule and memory networks predict outcomes for multi-attribute stimuli. The configural memory network (right) processes stimuli holistically, associating each with outcomes via Hebbian learning (strength λ), with exemplar-based inference during choice. The rule network (left) generates simple rules based on independent stimulus dimensions and tracks context features for rule modulation (strengths γ and ω, respectively). Rule and modulator attention weights (α, β) reflect confidence in associations (sum to 1). In the example, the simple antennae rule receives high rule attention (α_1_ = 0.94), while wings’ is the key modulator (β_2_ = 0.98). The Simple Rule could be square → Reward [A], and diamond → No Reward [B]’. If its predictions repeatedly mismatch with observed outcomes, CAL attributes this to the context feature that correlates with these successes and errors. Thus, the modulation updates are recency- and error-driven learning. If the learned feature (e.g., wings = two) modulation reverses the antennae rule, enabling optimum type II task performance.

CAL learns Simple Rules on independent stimulus dimensions (e.g., color), with associative updates driven by similarity-based generalization and dissimilarity-based extrapolation, where γ controls update precision.^13^^,^^14^ A key innovation was combining self-confirmatory Simple Rule learning with error-driven modulation.^42^ Modulation, governed by ω, is implemented as a recency-based tally of rule success and error, which enables context-dependent response gating,^18^^,^^19^ efficiently solving Type II problems (RB) via akin to spontaneous rule discovery (i.e., once the modulator is recognized accuracy jumps from guessing to optimal within very few trials). In contrast, in Type VI problems (U), CAL can only use its Configural Memory module to improve performance, which associates stimuli to outcomes trial by trial in a rather gradual fashion, thus, differentiating from RB in terms of learning trajectory. Formal details are provided in STAR Methods.

We adapted CAL for the current probabilistic stimulus-selection task, first, considering the following aspects as strategy unspecific. Regarding stimulus selection (choice phase), we assumed that CAL generally selects the stimulus that most strongly predicts reward, taking both modules’ predictions combined. Regarding the probabilistic feedback (outcome phase), we reasoned that participants expected 20% NoReward feedback by chance, and that an optimal solution maximizes the *probability* of reinforcement. Thus, feedback should be generally discounted or re-evaluated once a canonical solution is subjectively found, to prevent unlearning.^4^^,^^36^ CAL addresses this by ‘trusting’ the current predictions after three *confident* correct responses, switching to self-supervised learning. This deterministic error discounting, previously described as gradual cessation or annealing of learning,^64^^,^^113^^,^^114^ is captured in CAL’s feedback processing variables (RB: *v*_*t*_, U: λ_*t*_; see Table 1). Thus, regardless of the module, there only exist subjective Reward feedback in post-solution trials.

In pre-solution trails, however, feedback processing is module-specific in CAL. In the memory module (active in U), update strengths for chosen and unchosen stimuli are estimated depending on feedback (λ_*t*_). In the rule module (active in RB), after Reward feedback, CAL updates its rules only based on the chosen stimulus; after NoReward, CAL processes first the chosen then the unchosen stimulus, as suggested by the within-trial eye tracking data (see supplemental information). Crucially, in reward trials, CAL enhances rule extrapolation (success driven), while NoReward trials enhance rule modulation (error driven), which is captured by the derived parameter *v*_*t*_. Table 1 summarizes the relevant variables used in fMRI analyses.

Notably, taking CAL’s own trust in its rule solution in RB, and the reward-prediction strength of the chosen stimulus in U, are effectively highly similar to alternative measures typical in studies of economic choice, which often use the difference between the predicted values of both stimuli.^105^^,^^115^^,^^116^^,^^117^^,^^118^ In RB, on the one hand, CAL’s RB predictions are, with low rule trust, generally weak for both stimuli and thus the subjective difference between both stimuli is low (and vice versa with high trust). In U, on the other hand, our parameter estimates highlight that participants predominantly learned about the ‘rewarding’ stimulus (chosen after reward, unchosen after no-reward feedback), entailing that the difference between the reward-predictions of both stimuli is mainly driven by the subjectively rewarding stimulus (i.e., the currently chosen one). Note, the mean of the individuals’ correlation between choice confidence and reaction time was for RB *r* = −0.39 and for U *r* = −0.27 (both significantly smaller than 0 on average, *p* <0.001). While there seems to be the possibility that further correlations therefore could also reflect engagement in the task, it is common to view changes in reaction times rather as a consequence of learning and factors determining how difficult the task is or how confusable stimulus representations are.^119^^,^^120^

#### CAL-informed analyses

To identify theoretically informed differences between RB and U, we defined a second first-level model. Each onset regressor was modulated with individual model-derived parameters (Table 1), i.e., choice of RB with Rule Confidence, choice of U with Memory Confidence, outcomes of RB and U with the task specific Reward Influence. Due to the highly task-specific CAL estimates (i.e., its rule module is formally incapable of solving U, and the memory module is formally incapable of predicting sudden rule discovery after a period of guessing; Figures 2 and 3), we only used the estimates stemming from the rule module for RB, and the memory module for U. After visual inspection of a single-trial onset analysis conducted for visualization purposes, we removed the very first trial in each of the five runs (i.e., modeled separately as nuisance), as we observed substantially greater activity in all 16 inspected peak voxels for the choice onset compared to the following trials in each of the five runs, which would bias the model estimates. Movement parameters were included as nuisance regressors. All CAL predictors were z-standardized within participants (*M* = 0, *SD* = 1) to remove variance stemming from between-participant strength differences, which not necessarily translate to between-participant variations in BOLD activity.

On the second level, we identified common areas involved in both learning problems using a conjunction analysis on the parameter estimates of the task-specific model-derived parametric regressors. To identify task specific areas, we applied exclusive masking with the conjunction (liberal threshold p ¡ .1 uncorrected) instead of a differential contrasts between the two task-specific regressors, because the non-matching model-informed regressor (e.g., memory module in the RB task) would have resulted in a comparison against a meaningless parameter.

Note, we also conducted t-tests as control analyses after shuffling the participants’ model predictions between individuals (cet. par. with the original analyses) for all peaks reported in Tables S2 and S3. In particular, 82 t-tests were conducted, two in each of the areas identified by conjunction analyses and one in each area exclusively involved in rule-based and unstructured category-learning. 11 of the t-tests (13%) revealed that the shuffled regressors results not in significantly smaller parameter estimates, 9 of them in memory confidence which is not surprising as it reflects Hebbian learning which increases with repeated exposure to the stimuli and correlates therefore across participants. Overall the results of this control analysis support the participant-specificity of our modeling approach.

Correction for multiple comparisons was done cluster-wise FWE *p* <0.05, cluster inducing threshold *p* <0.001,^121^ at the whole-brain level and within predefined anatomical regions of interest based on previous learning studies. In particular, the hippocampus, the triangular IFG, caudate, putamen and N. accumbens have been repeatedly reported to be involved in category learning.^2^^,^^23^^,^^24^

#### CAL parameter estimation method

We applied CAL separately to each individual and task (RB versus U) on the exact same trials the participants saw, estimating six parameters in each (rule learning: γ, ω, ρ^I^, ρ^A^; memorization: λ, λ_*R*_, λ_*NR*_). For each task, we used both pre-training (deterministic trials) and main task, without free parameters between both, estimating the parameters via the differential evolution R-package DE Optim.^103^^,^^122^ The respective lower limits of the parameter space were -4,-5,0,0,-5,-3,-3, and the upper limits were 4, 5, 2, .2, 5, 3, 3. For example, the range -4 to 4 for γ are those values on which CAL generates behavioral variations in the given task, but not beyond these values. DE optimization randomly samples values in iteration *n*=1 and mutates the values with the highest likelihood to iteration *n*+1, which is suited to estimate parameters with non-linear outcome dependencies. We ran 500 iterations for each participant. Note, the solutions to some degree depend on sampling variations, and there might be other parameter sets resulting in better fits. However, exploring different random seeds led to virtually the same results.

Note, that setting a low limit of 0 for ρ^I^ represents a theoretically directed hypothesis, which assumes that gaining an immediate reward (coded as +1 in the model), relative to no-reward trials (coded as 0 in the model), can only reduce the function value of *v*_*t*_ (Equation 10), which translates to more precise rule updates (i.e., γ_*t*_ decreases/narrows the generalization function; Equation 12), while strengthening the Simple Rule priors that led to this reward (Equation 13), but weakening modulatory updates (Eq.’s 19 & 20). Correspondingly, positive ρ^A^ lead to increasing values of *v*_*t*_ with accumulated rewards, which in turn broadens generalization over time, but also leads to generally stronger modulation updates (akin to gaining cognitive control over time). In Schlegelmilch et al. (2023), we have shown that these assumptions successfully predict behavior in probabilistic reward learning and risky gambles. Crucially, the latter include situations in the loss domain (i.e., negative reward, e.g., coded as -10), meaningfully changing the sign of the *v*_*t*_ function’s dependencies. Thus, in order to provide non-arbitrary predictions, the *sign* of ρ^I^ and ρ^A^ need to be fixed, and we here consequently consider positive values for corresponding interpretations, consistent with previous conclusions. Further note, in post-solution trials (self-supervised learning), we fixed λ_*t*_ to -3 and -5 for chosen and unchosen stimuli, respectively, because memory-update strength is not identified when performance is at ceiling already.

As objective criterion we calculated the summed binomial -LogLikelihood (-LL) of the trial-wise individual’s data given the choice predictions, in a first step. However, since participants performed the pre-training only until reaching 15 consecutive correct responses while completing 160 main-task trials, the former contributed a lower number of trials to the overall LL. For the total LL calculation, thus, we multiplied the pre-training LL with the ratio of trials between pre-training and main task. For example, with 80 pre-training trials, the ratio would be 2, thus, multiplying the pre-training LL by 2 (i.e., as if 160 trials were performed), ensuring that the main task did not dominate the estimation procedure due to the higher number of trials. This implies, that the parameter estimates used in the CAL-informed fMRi analyses are also informed by the pre-training task, supporting their reliability. Note, however, especially the three memory parameters result in high flexibility of the memory module, and we applied three constraints for reliable estimation.

Besides optimizing an individual’s task-specific parameters based on both pre-training and main task (i.e., identical six parameters; see Figure S5 for pre-training learning curves), first, (1) we applied (linear) parameter regularization.^123^ This means, free parameters can only become effective if they contribute to explaining variance (e.g., different-from-0-estimates for the difference estimates of ρ^A^, λ^R^, λ^NR^, only if they improve the fit). For example, we added the estimate of ρ^A^ to the overall LL of the pre-training (and to the main task LL, see below), such that higher values decreased the fit statistic. This means, ρ^A^ remained 0 if the explained variance (lower -LL) by larger parameter values did not compensate for impoverishing the fit statistic (for the unconstrained difference parameters, e.g., λ^*R*^, we added the absolute deviation from 0). However, taking the computational flexibility of the memory module into account, we applied twice the weight to λ than to all other parameters (i.e., small changes in λ have relatively large impact). Note, CAL’s γ parameter is already formally regularized in the mechanistic model hypotheses, for which we applied no regularization (i.e., it can only become effective if participants show behavior akin to a rule solution). This greatly facilitates immediate conclusions, without requiring factorial model comparison. Consequently, we only consider the process predictors mainly active in each task, that is, we selectively use CAL’s rule module parameters in RB, and its memory module parameters in U for analyses.

Second, (2) as another regularization constraint we take the natural relation between LL, behavioral accuracy (data) and model fitting into account. For example, consider a participant solving the RB main task in 10 trials, compared to another one solving it in 100 trials. Statistically, the former is nearly uninformative in model fitting, because fitting highly accurate behavior is easy for any model designed to solve the given task (rule or memory in RB). In contrast, the challenge for learning models actually is to explain variance before arriving at a task solution (i.e., in predicting decision errors), which is more informative regarding parameter estimation. We took this aspect into account by multiplying the above regularization penalties in each pre-training and main task by the corresponding average accuracy in percent (based on the given number of trials). Thus, lower accuracy leads to lower parameter penalties. Third, for the same reason, (3) we enforced CAL to search for a rule solution by fitting both its overall choice prediction (including both rule and memory modules) *and* its rule module’s (only) choice predictions to the data. This means, all-together, the rule module becomes effective in explaining variance if behavior reflects applying a rule, and the memory module only becomes effective if it explains additional variance beyond the rule predictions. Therefore, the parameter estimates in Figure 2 can be interpreted as providing evidence, that RB involves hardly any variance explained by memorization beyond CAL’s rule learning. Vice versa, there is hardly any variance explained by rule learning in the U task. Note, however, that two individuals, indeed, were best described by rule learning even in the U task (see strong γ, Figure 2I in U task), also showing choice behavior corresponding to a single-dimensional rule (not shown; e.g., when red hat, then rewarding, despite not being better than chance according to feedback throughout). However, since the vast majority of participants showed behavior in line with the task instructions, we did not further exclude any participants.

In sum, our primary goal was to obtain reliable parameter estimates, justifying the above fitting constraints. They rule out that the process estimates are biased due to several sources of noise. The success of this method is corroborated by CAL’s performance in terms of approximating behavior in both pre- and main task with a single set of parameters, strong parameter correlations with behavioral variables (e.g., trial solved *t*_*s*_), and process data that CAL was not optimized on (attention measures), and eventually its fMRI correlations.
