# Supplementary material for: Neurocomputational modeling of rule abstraction and memorization during probabilistic stimulus-reward learning
Source: iScience. 2025 Nov 14;29(1):113950. doi: 10.1016/j.isci.2025.113950 (PMC12834106; doi:10.1016/j.isci.2025.113950)
Supplement: Document S1. Figures S1–S7 and Tables S1–S3 [file mmc1.pdf]

iScience, Volume *29*

Supplemental information

Neurocomputational modeling of rule abstraction and memorization during probabilistic stimulus-reward learning

René Schlegelmilch, Alina Dinu, Gina Joue, Jan Gläscher, and Tobias Sommer

Supplemental Materials

René Schlegelmilch[1], Alina Dinu[2], Gina Joue[2], Jan Gläscher[2], and Tobias Sommer[2]

[1]University of Bremen

[2] Institute of Systems Neuroscience, University Medical Center Hamburg-Eppendorf, Germany

Supplemental Materials

Contents

Supplemental Materials 2

Main Task – Humanoid Stimulus Illustration 4

Pre-training – Stimulus Illustration 4

Eye-Tracking Analyses 5

Gaze Cascade During Choice 5

Gaze Cascade During Outcome Processing 7

Training Data and CAL Fits 9

fMRI Results Tables 12

Formal Description of CAL 18

Category predictions 18

Stimulus representation 18

Configural memory 18

Rule predictions 19

Contextual modulation 19

Category probabilities 20

Stimulus preference (Novel Hypothesis) 20

Learning 21

Reward function 21

Rule learning 22

Learning modulation 25

Modulation error reset 26

Attention learning 26

Updating of Two Stimuli (Novel Hypothesis) 27

Solving RB and Self-Supervised Learning (Novel) 28

Configural Memory 29
Interpretation of CAL predictors 30

Bibliography **34**

Main Task – Humanoid Stimulus Illustration

As highlighted in the main manuscript, all participants solved both RB and U problems on separate day sessions. The butterfly stimulus set is reported in the main manuscript. Fig. 1 shows the second stimulus set (humanoids).

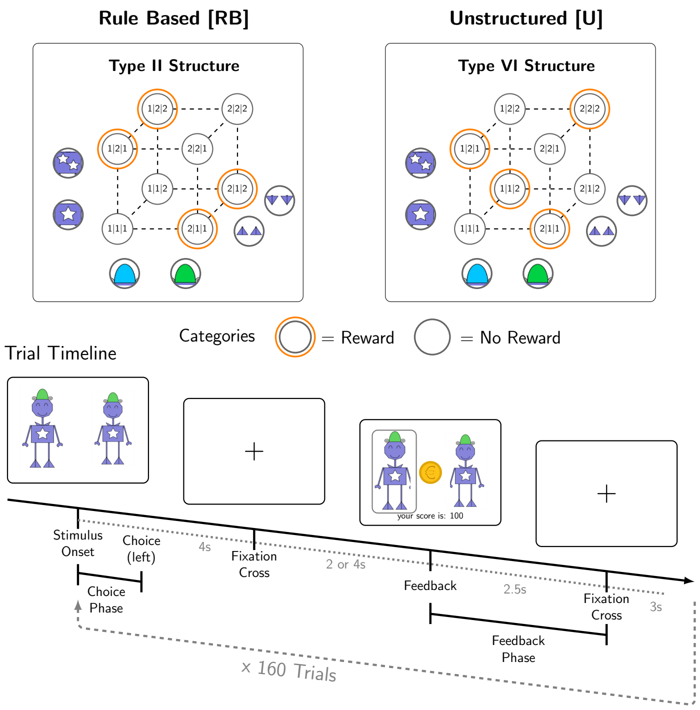

Figure 1. Task Design and Procedure. **(Top)** Problems Type II (Rule Based [RB]) and Type VI (Unstructured [U]). Numbers = coordinates of corresponding binary features (belly, hat, shoes). Orange circles = reward category. **(Bottom)** Trial procedure: (Stimulus Onset) Participants predicted the reward-category stimulus. Feedback reflected the canonical categories in 80% of 160 trials (probabilistic). Choice and Feedback Phases refer to eye-tracking analyses. Seconds refer to between-slide intervals.

Pre-training – Stimulus Illustration

Figure 1 shows the two separate sets of eight stimuli for the pre-training task. Each set consisted of geometrical figures with three dimensions. The first set contained figures, which varied in color (red or blue), shape (square or triangle) and filling (filled or not filled). The second stimulus set contained figures which also varied in color

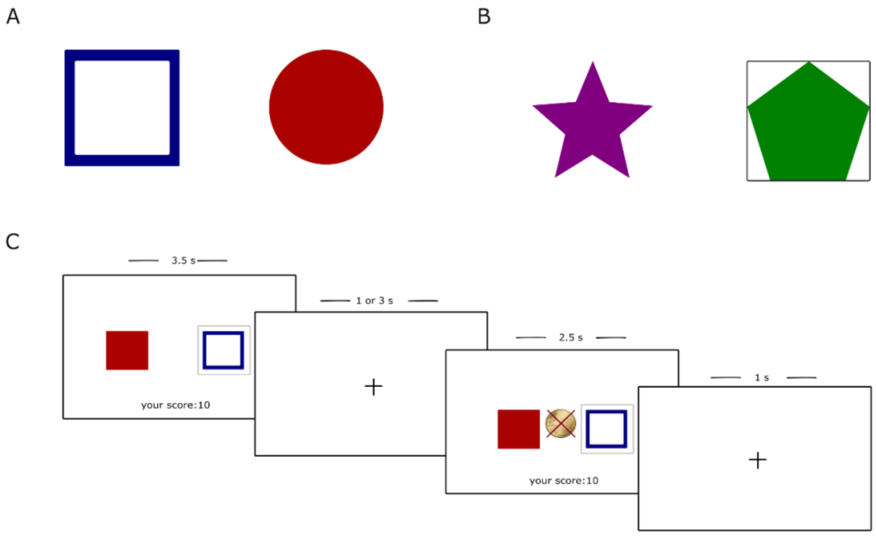

Figure 2. Deterministic pre-training task. (A) Representative pair for the deterministic rule-based training task. Stimuli differ in color (red or blue), shape (circle or square) and filling (filled or not filled). (B) Representative pair for the deterministic stimulus-based task. Stimuli differ in color (green or purple), shape (pentagon or star) and contour (with or without). (C) Example trial. The numbers on top of each screen indicate its presentation duration in seconds.

(green or purple) and shape (star or pentagon) but had contour as third dimension (with or without contour).

Eye-Tracking Analyses

In all analyses below, we use the fixations exclusively made to the two stimuli (i.e., cleaning of other fixations before). When counting fixation sequences, we ignore fixations that occurred in-between fixations of the two stimuli, if those were somewhere else on the screen. Thus, the overall probability of fixating the chosen and unchosen stimuli always sums to 1 in the following analyses.

Gaze Cascade During Choice

In preferential choice tasks a common observation is that of the so-called gaze-cascade effect (e.g., Glaholt & Reingold, 2011; Shimojo, Simion, Shimojo, & Scheier, 2003), describing that attention is biased to the chosen stimulus, briefly before

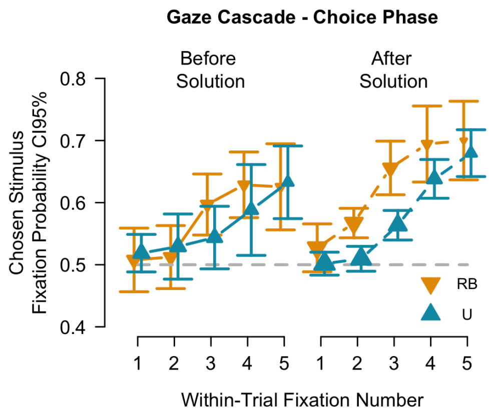

Figure 3. Choice Phase Gaze Cascade. Likelihood of fixating the eventually chosen stimulus (y-axis), depending on fixations (x-axis) before termination (last and previous fixations) within a given trial, before and after the solution in each task. Error bars = 95% CIs of the mean of the individuals' mean probabilities across trials. Symbol sizes reflect number of available participant observations.

making a choice. In our task, we observed the same phenomenon. Fig. 3 shows, the relative proportion of trials (y-axis) in which the first, second, third, fourth and fifth fixation (x-axis) fell on the eventually chosen stimulus (averaged within, then across participants, before and after solving the task). As can be seen. Before before the solution in RB, participants made about two fixations before focusing on the chosen stimulus, but only one after the solution. In contrast, in U, they took about three fixations before, and two fixations after the solution, but the trend was more pronounced after the task was solved.

To test this, we entered the participant's average fixation likelihood as dependent variable in a hierarchical mixed model, with fixation number (first five fixations, factorial), task (RB vs. U), and phase (pre vs. post-solution) as factorial predictors. As random effects we specified by-participant random intercepts. We found a significant main effect of task $\chi(1) = 12.28$, $p < .001$, a significant main effect of phase, $\chi(1) = 6.71$, $p = .01$, and a significant main effect of fixation number, $\chi(4) = 141.61$, $p < .001$. Moreover, we found a significant interaction between task and phase $\chi(1) = 4.04$, $p = .044$. All other interactions were non-significant ($p > .15$). As Fig. 3

indicates, the significant interaction between task and phase implies, that participants more likely fixated the chosen stimulus in RB compared to U after the task was solved, which was weaker before the task was solved. The absence of a three-way interaction with fixation number implies that the speed of increase (slope) in the likelihood was nonetheless the same in RB and U.

In essence, thus, this pattern speaks for the idea that stimulus comparison becomes quicker after solving the RB task. This interpretation would be in line with previous suggestions that the gaze-cascade effect is associated to confirmatory processes of response execution (although it does not rule out continued processing of the chosen stimulus; e.g., Glaholt & Reingold, 2011). Accordingly, in adjusting CAL's process assumptions, we accommodate for this empirical result by including an initial stimulus comparison, in which CAL calculates the reward probabilities of each stimulus, of which we take the winner for making the corresponding behavioral predictions. Furthermore, we assume that there are no further evaluations of the chosen stimulus to render the final choice.

Gaze Cascade During Outcome Processing

As reported in the main manuscript, we also found a cascade effect in stimulus processing during the outcome phase. Of interest is how participants switched between both stimuli, and we focus on the probability of fixating the chosen stimulus, depending on the within-trial forward sequence of fixations, to motivate the adjustment of CAL's mechanistic assumptions of its rule learning module.

Fig. 4 shows, in reward trials before the solution, participants almost exclusively fixated the chosen stimulus, only slightly changing in probability over successive fixations compared to no-reward trials. After receiving error feedback, however, participants quickly switched to the stimulus which, according to feedback, would have led to reward (i.e., the unchosen stimulus). Interestingly, this trend seemed equally strong in RB and U, and in both the trend changed once the task was solved. In the latter case, most fixations went to the chosen stimulus. And as can be seen from the

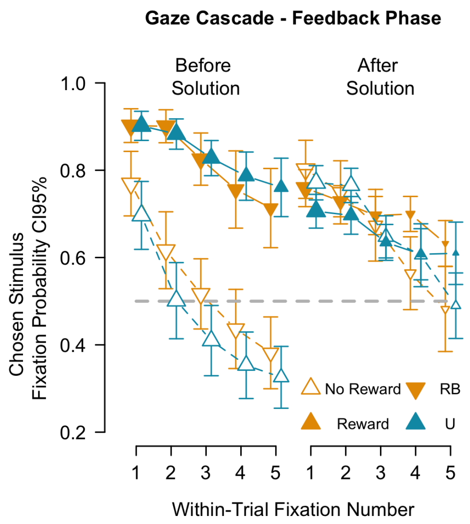

Figure 4. Feedback Phase Gaze Cascade. Likelihood of fixating the chosen stimulus (y-axis), depending on feedback (reward=filled vs. no reward=hollow symbols), over successive fixations (x-axis) within a given trial, before and after the solution in each task. Error bars = 95% CIs of the mean of the individuals' mean probabilities across trials. Symbol sizes reflect number of available participant observations.

symbol sizes (reflecting the number of available observations), they made fewer fixation in general on each of the stimuli (also indicated by the wider confidence intervals).

We applied the same model as in the previous section, including fixation number, task, phase, and the feedback (reward vs. no reward), and by-participant random intercepts, but also including random slopes for task, feedback and phase (such more complex structures caused singularity issues in the previous analyses, but not in the current one). We found a main effect of task, $\chi(1) = 4.31$, $p = .038$, indicating a slightly higher fixation probability in RB compared to U in general. There was also a main effect of fixation number,$\chi(4) = 396.24$, $p < .001$, indicating that with ongoing fixations, fixating the unchosen stimulus became increasingly more likely in general. There also was a significant effect of feedback, $\chi(1) = 45.91$, $p < .001$, such that the unchosen stimulus was more likely being fixated in no-reward compared to reward trials.

Furthermore, there was a significant interaction between phase and fixation number, $\chi(4) = 18.3$, $p < .001$, between fixation number and feedback, $\chi(4) = 80.11$,

$p < .001$, task and feedback, $\chi(1) = 298.00$, $p < .001$, a three-way interaction between task, phase and feedback, $\chi(1) = 17.95$, $p < .001$, and fixation number, phase and feedback, $\chi(4) = 15.75$, $p = .003$. There was no significant four-way interaction, and the remaining effects were non-significant as well ($p > .1$). While some of these interactions seem difficult to interpret, the straight-forward take-away message from this analysis is that stimulus processing generally reflected a strong switch from chosen towards unchosen stimulus in no reward trials but rather selective processing of the chosen stimulus before the solution, regardless of the task.

Overall, thus, it seems that the process data displays a meta-strategic information search depending on feedback and task solution (t_s), which we took into account in CAL. Overall, first, we assumed that the chosen stimulus is taken as rewarding after solving the task. Second, in RB, we assumed that CAL, in reward trials, only processes the chosen stimulus, but in error trials both the chosen and unchosen stimulus (in this order), which consequentially also translates to corresponding stimulus-attention estimates together with the processing strength estimates (i.e., v_t; see main manuscript and formal description below). In the U task, however, we take a different approach, as CAL's memory definitions are non-sequential but more descriptive. Here, we estimated how strongly each stimulus is encoded in each trial, which should mirror the dwell times on each stimulus in terms of cross-validation.

Training Data and CAL Fits

Before participants performed the main task in the scanner, they were familiarized with the RB and U problems in a pre-training task. Both problems were of the same type in pre-training and main task (e.g., only RB). However, in the pre-training task, there was deterministic feedback, and the training phase stopped once reaching a learning criterion (to generate the learning curves in the graphs below we treat every trial after that as solved, up to 160; in fitting we only used the available trials). Moreover, participants knew via instruction in RB pre-training, but not in the main task, which dimension was irrelevant, which we implemented in CAL by letting it ignore

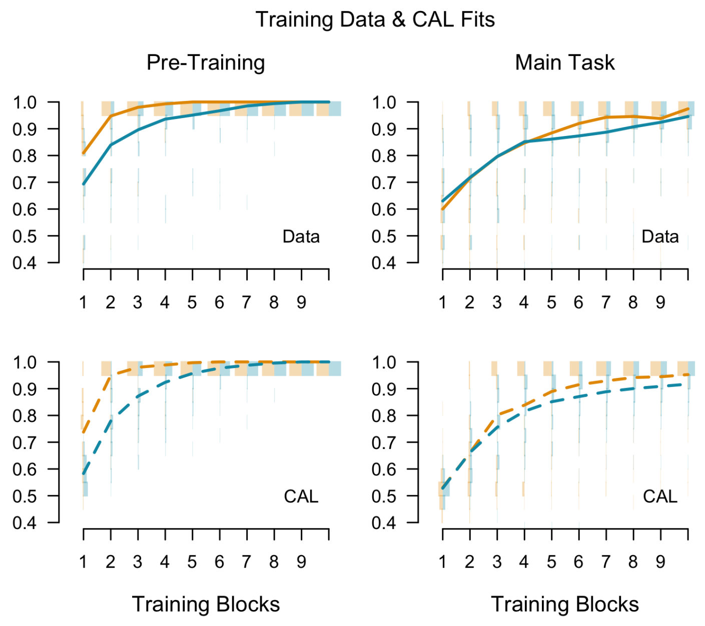

Figure 5. Learning Curves and CAL Fit. Average accuracy (canonical categories; y-axes) for Pre-Training and Main Task (top) and corresponding CAL fit (bottom), over training blocks (x-axes, 16-trial bins). Orange= RB, blue = U. Histograms in background reflect distribution of individual accuracy.

the irrelevant feature dimension. Theoretically, thus, using both tasks in fitting ensures that the CAL assumptions that concern learning under probabilistic feedback did not lead to implausible predictions under conditions of deterministic feedback (as studied in Schlegelmilch et al., 2022).

As can be seen in Figure 5 (top left), under these conditions the learning curves differed between RB and U during pre-training, and CAL well accomodated this despite using the same set of parameters for both in each problem. One minor deviation between the data and CAL concerns the fact that some participants reached 100% accuracy after very few trials, which is not achieved by CAL (i.e., slightly lower average learning accuracy than in the data in block 1), equally in RB and U, but which seems negligible. Interestingly, the difference between RB and U accuracy in pre-training with deterministic feedback would be expected from previous studies on these problems (e.g., Shepard et al., 1961; Nosofsky, Gluck et al., 1994), and is predicted by CAL in this

task. However, since participants knew which feature was irrelevant in RB, it is unclear whether or not the same result would be observed if this was not the case under explicit rule instructions (slower rule discovery by searching for the irrelevant dimension). However, that probabilistic feedback drives these different patterns in pre-training and main task seems likely, if rule discovery is based on modulation of Simple Rules as assumed in CAL. That is, spontaneous rule discovery there is due to error-based structuring of Simple Rules (conditional hypotheses), which requires reliably observing Simple Rule errors in the same context (e.g., antennae rule applies if one circle, but not when two circles). In case of probabilistic errors, already suspected modulators are quickly unlearned, which slows down corresponding attention learning mechanisms in CAL (β) to focus on the true modulator. Thus, finding this solution requires that the rule modules predictions are correct in a few consecutive trials to establish confident predictions and to decide that the task is solved, which happens more sporadically due to randomness, than with deterministic feedback.

fMRI Results Tables

Table 1

Model-free fMRI Results.

**Choice Phase**	Region	MNI coordinates	Z
**RB > U**	triangular inferior frontal gyrus	-54 28 4	5.17
		50 38 0	6.66
	middle frontal gyrus	-30 28 46	3.91
		14 54 26	5.72
		34 28 46	4.47
	superior frontal gyrus	-26 50 28	4.92
		14 54 26	5.72
		16 18 60	5.74
	supramarginal and angular gyri	-58 -46 36	6.92
		58 -44 40	5.96
	middle temporal gyrus	58 -44 8	6.76
		62 -22 -8	6.20
	anterior hippocampus	-32 -22-16	3.68
		24 -10 -18	4.90
	vmPFC/ACC	-6 38 -4	5.35
	precuneus	-4 -40 46	5.06
	posterior insula	-40 6 -6	4.80
		40 4 -8	5.41
	putamen	-30 -4 -8	4.81
		28 -2 -8	4.83
**U > RB**	anterior insula	-30 20 4	5.44
		32 26 0	5.29
	caudate head/putamen	-8 6 6	5.21
		10 6 8	4.45

	SN/VTA	6 -18 -12	4.42
		-2 -22 -20	3.75
**Outcome Phase**	Region	MNI coordinates	Z
**RB > U**	triangular inferior frontal gyrus	48 38 0	4.91
	supramarginal gyrus	-58 -44 36	5.64
		56 -44 26	4.25
	superior parietal lobe	-34 -48 42	5.30

Significant peak voxels corrected for multiple comparisons across the whole scan volume. For the outcome phase the contrast U > RB did not result in any significant voxels.

Table 2

Model-based fMRI Results: Choice Phase.

Region	MNI coordinates	Z-value
Conjunction RB & U (positive)		
vmPFC	-12 46 0	4.70
anterior cingulate	-6 24 24	5.19
posterior cingulate	6 -32 46	6.03
middle frontal gyrus	-34 42 28	4.36
	36 50 24	4.12
supramarginal gyrus	-64 -46 28	4.91
	56 -42 34	4.28
middle temporal gyrus	56 -26 -4	5.09
posterior hippocampus	-22 -38 -2	4.27
	22 -36 0	3.79
	32 -22 -16	3.56
precuneus	-10 -66 34	4.95
	16 -64 32	4.76
putamen	-32 -12 2	4.55
anterior insula	-42 2 -2	4.78
temporal pole	-48 12 -6	4.85
	50 12 -8	4.89
RB exclusive (positive), masked with conjunction ($p<$ .1 uncorrected)		
planum polare	56 6 -8	4.72
U exclusive (positive), masked with conjunction ($p<$ .1 uncorrected)		
caudate (head, body)	-14 16 2	5.00
	16 14 6	4.96
	-16 10 16	4.50
	18 4 18	4.56

putamen	20 12 2	5.77
	-24 10 -8	4.91
superior parietal lobe	-22 -42 66	4.38
	30 -66 54	5.65
angular gyrus	-32 -70 50	5.63
	32 -66 46	5.37
precentral gyrus	-50 -8 44	6.00
early visual cortex	-34 -90 12	5.58
	30 -88 14	6.73
	-10 -96 14	5.93
	20 -92 22	5.62
cerebellum	28 -66 -26	5.84
RB exclusive (negative)		
triangular inferior frontal gyrus	-44 42 -4	4.55
middle frontal gyrus	-38 22 26	4.50
fusiform gyrus	30 -50 -16	4.41

Significant peak voxels corrected for multiple comparisons across the whole scan volume. In addition, peak voxels are reported corrected for multiple comparisons within regions of interest svc.

Table 3

Model-based fMRI Results: Outcome Phase.

Region	MNI coordinates	Z-value
Conjunction RB & U (positive)		
N. accumbens	-12 12 -6	3.18
RB exclusive (positive), masked with conjunction (p< .1 uncorrected)		
triangular inferior frontal gyrus	-50 28 8	6.08
caudate (tail, body, head)	-16 8 20	5.21
	-10 -18 2	5.18
	18 8 20	6.10
	12 22 -4	5.19
	18 -10 26	3.54
anterior hippocampus	-34 -18 -14	4.37
posterior hippocampus	36 -28 -12	5.11
	28 -36 0	3.83
supplementary motor cortex	-4 2 64	5.43
middle frontal gyrus	-26 20 46	4.94
middle temporal gyrus	-62 -36 0	4.90
	64 -24 -6	3.90
occipital pole	-12 -96 14	5.77
U exclusive (positive), masked with conjunction (p< .1 uncorrected)		
vmPFC	-12 46 0	4.49
anterior hippocampus	-30 -8 -22	3.48
posterior hippocampus	-32 -26 -16	3.52
central operculum	42 -22 28	4.48
	-42 0 10	4.16
	38 -4 -4	4.40
parietal operculum	-52 -32 22	4.44

precentral gyrus	-30 -24 56	4.36
postcentral gyrus	22 -34 62	4.00
U exclusive (negative)		
orbital inferior frontal gyrus/anterior insula	44 28 -8	4.86
middle frontal gyrus	42 14 34	4.72
medial superior frontal gyrus	6 28 40	4.00
angular gyrus	48 -56 38	3.75

Significant peak voxels corrected for multiple comparisons across the whole scan volume. In addition, peak voxels are reported corrected for multiple comparisons within regions of interest svc. It is noteworthy that the negative correlation of activity with Feedback Processing in U of the the middle frontal and angular gyri might be bilateral as also correlations on the left side were observed although not significant after correction for multiple comparisons ([-42 14 38], Z = 3.80; [-38 -56 42], Z = 3.63).

Formal Description of CAL

Category predictions

Stimulus representation. CAL separates the stimulus S into feature dimensions m (e.g., color and size), which we assume to exist from the beginning. Each dimension has its own sets of nodes aligned by the feature values they represent (e.g., small =1, 2=medium, 3=large). For binary dimensions we assume two nodes, as in the current study. However, those are re-scaled to 0 and 1 within the CAL mechanisms (e.g., for antennae, square=0, diamond=1), to calculate the similarity functions below based on the minimum and maximum value observed. Formally, physical values (x_{mi}) are unitized to σ_{mi}. The current task has $M = 3$ binary-valued dimensions, where the binary values are called nodes i. [1]

$$\sigma_{mi} = \frac{x_{mi} - \min(x_m)}{\max(x_m) - \min(x_m)} \tag{1}$$

Configural memory. Stimulus S activates previously stored instance representations y via psychological distance d_y. They have outcome associations (to A=Reward, to B=No Reward) with strengths h_{yk} (initialized to 0).

The normalized values are used to compute the sum of distances between the current stimulus S to each stored instance:

$$d_y = \sum_m |\sigma^S_{mI} - \sigma^y_{mi}| \tag{2}$$

The total associative strength h_{yk} of each instance y is transformed to

$$c_y = .5 \cdot \exp(-.25 \cdot \sum_k h_{yk}) \tag{3}$$

The values of .5 and .25 are scaling constants. c_y reflects a similarity weight to calculate the overall sum of category activation a_k. Formally,

[1] Throughout, a lowercase subscript (e.g. i) denotes the set of possible values of that index, while an uppercase subscript (e.g. I) denotes a specific value within that set, usually the selected or active unit currently visible on screen for a given stimulus.

$$a_k = \sum_y \exp\left(- \frac{d_y^2}{2 \cdot c_y^2} \right) \cdot (.1 + h_{yk}) \tag{4}$$

The memory-based prediction H_K is then calculated as:

$$H_K = \ln\left(\frac{a_K}{\sum_{k \neq K} a_k} \right) \tag{5}$$

This is done for both presented stimuli equally.

Rule predictions. Each dimension node i on a dimension m is associated to its own set of outcome nodes o (A=Reward and B=No Reward) with strength w_{mio} (initialized to $1/(M+1)$, M = number of dimensions).

On each dimension m, the evidence ratios of the activated node [I]-to-outcome[o] associations yield dimension-specific rule predictions r_{mIo}, called Simple Rule predictions, calculated as log 'posterior odds' for a specific outcome O divided by the sum of associations to other outcomes. Formally,

$$r_{mIO} = \ln\left(\frac{w_{mIO} + .1}{\sum w_{mI(o \neq O)} + .1} \right) \tag{6}$$

However, the prediction of outcome O is not the final response, but is passed to a gating mechanism of contextual modulation, which gives the consequential prediction of category k for the given stimulus.

Contextual modulation. For the modulators we use the notation j nodes and n dimensions (instead of i and m) for clarity, but they refer to the same stimulus features with $m \sim n$ and $i \sim j$. The j gating nodes of a modulator dimension n register the accuracy of the simple rule predictions (except if coming from dimension $m = n$). For re-gating the rule to response k, the active node J is taken (e.g., wings=one circle if currently visible and the evaluated simple rule concerns the antennae):

$$z_{mIk} = \alpha_m \sum_n \sum_o r_{mIo} \cdot 1/(1 + \exp(-v_{mnJok})) \tag{7}$$

The modulator nodes v_{mnjok} (see below) are initialized with .5 for matching and -.5 for mismatching outcome-response associations. The attention parameter α_m is initialized to $1/M$ (sum to 1).

Category probabilities. The category response k for a given stimulus S is calculated as

$$p(k|S) = \frac{1}{1 + \exp(-2.5 \cdot [H_k + R_k])} \tag{8}$$

where H_k is the memory-based evidence, and R_k the rule-based evidence. The rule module's prediction R_k is defined as:

$$R_k = \sum_m z_{mk} \tag{9}$$

A scaling scaling constant of 2.5 is used instead of a free response-determinism (or heat) parameter, because a free scaling parameter would hinder identifying CAL's (or any other model's) learning rate parameters as low response scaling values (leading to pure guessing) basically leave infinite degrees of freedom regarding the variations of the unscaled predictions, if those become inconsequential.

In the original CAL version (Schlegelmilch et al., 2022), we assumed that $\sum_m z_{mk}$ is discounted if the memory module makes a strong prediction (i.e., automatic intervention based on memory), which we excluded here, for the reasons explained in the main manuscript (section Memory storage under probabilistic feedback).

Stimulus preference (Novel Hypothesis). When presented with two stimuli, CAL calculates the above predictions for each stimulus separately in a first processing stage, which gives two predictions for each stimulus, namely, how likely the left stimulus yields reward, for example, p(Reward|Left)=.7 and p(No Reward|Left)=.3, and likewise for the right stimulus, p(Reward|Right)=.2 and p(No Reward|Right)=.8. We then take the plain average of these predictions with A= Reward, B=No Reward, L=Left stimulus and R = Right stimulus, and $p'(A|L) = (p(A|L) + p(B|R))/2$, and likewise for $p'(A|R) = (p(A|R) + p(B|L))/2$, and define the left stimulus as 'chosen' if $p'(A|L) > p'(B|R)$, otherwise the right stimulus. However, the calculation does not necessitate that both stimuli render the same prediction. For example, it could be that CAL predicts $p(A|L) = .8$ and $p(B|L) = .2$ for the left stimulus (note that both predictions are symmetric, sum to one), but for the right stimulus $p(A|R) = .55$ and $p(B|R) = .45$. This can happen, for example in the U task, if CAL hardly observed the

right stimulus before, or associated the stimulus, during prior learning, equally strongly to both reward and no-reward categories due to probabilistic feedback. In this case, however, CAL would choose the left stimulus, and we take its corresponding prediction of $p(A|L) = .8$ as probability of a left choice and $p(B|L) = .2$ as probability of a right choice (which is equal to $1 - p(A|L)$). Thus, the strength of these predictions, in U, depends on which stimuli CAL encodes more strongly in memory, and in RB, which rule and modulator associations each stimulus activates.

In model fitting, we then take the probability-strength predictions based on CAL's chosen stimulus to be applied to the participant's choice data. For example, we coded the 'left' decisions for a participant as yes, no, yes no (i.e., as 1,0,1,0), and apply the corresponding prediction strengths in CAL (e.g., .9,.3,.7,.2), where values higher than .5 imply that the left option was chosen by CAL (taking $p(A|L)$), and values lower than .5 imply that the right option was chosen by CAL (taking $1 - p(A|R)$).

Learning

Reward function. Our general reward hypothesis that affects the below explained updates is defined as v_t, reflecting variable reactions to reward over trials t, formally,

$$v_t = -r_t \rho^I + \sum_{1}^{t} r_t \rho^A \tag{10}$$

The value r_t reflects the current payoff, weighted by the salience weight ρ^I. We set $r_t = 1$ if CAL chose the stimulus for which the feedback signaled reward, and $r_t = 0$ otherwise in a given trial. The choice is defined as in the previous section (chosen stimulus). In fitting, we estimate ρ^I individually, but constrained to take positive values to not cancel out our hypothesis that a current reward reduces v_t and omitting a reward increases v_t, as indicated by its sign. The second term in Equation 10 reflects accumulated rewards over time, again weighted by a salience parameter ρ^A, estimated in fitting, again constrained to values larger than 0. The signs in the formula imply, that accumulating a number of gains gradually increases v_t over time. In the later functional applications, this has the consequence that trial-wise variations of r_t will

have increasingly less impact over time.

Additionally, since we included a novel hypothesis that CAL switches to self-supervised learning at some point (see below), this implies that after this time point (prediction = reward), $r_t = 1$ in any case. Thus, v_t is identified by behavioral reactions to feedback before solving the task including a reduction of the impact of r_t depending on accumulated gains, and afterwards rather reflects a continued hypothetical increase of v_t due to further accumulated gains, which, however, more quickly accumulate than before solving the task in RB. In the U task, this hypothesis will have no practical effect, due to not contributing to solving the unstructured problem, which participants knew, which we took into account in the fitting procedure.

Rule learning. In the following description of the learning processes we do not use the notation of A (reward) and B (no reward) categories, since the mechanisms are defined in terms of which category was actually observed for a given stimulus, which could be both A or B. We first outline the updates for the stimulus chosen by CAL (e.g., left). Let the *present* (observed) category be P, and the *absent* (unobserved) category be $\bar{P}$. The associations to category P are updated via excitatory generalization (similarity), and those to $\bar{P}$ are updated via contrasting (dissimilarity). Their general magnitude can vary *across* the dimensions through Ω_m, explained later.

Excitatory generalization. The associations of the dimension nodes to category P (w_{miP}) are updated by adding Δw_{miP} (Gaussian decay). Formally,

$$\Delta w_{miP} = \Omega_m \cdot \exp\Big(-\frac{|\sigma^S_{mI} - \sigma_{mi}|^2}{2 \cdot \exp(\gamma_t)^2}\Big) \cdot \Big(\phi_t + \exp\Big(-\frac{\sum_{\bar{p}} w_{mi\bar{p}}}{w_{miP}}\Big)\Big) \tag{11}$$

γ_t governs the width of the similarity decay with psychological distance. Lower values of γ_t narrow the update function.

Weighting the similarity function by the ratio of its existing associations (w_{mio}), represents belief updating and reduces the update for nodes that only weakly predict P, similar to a Bayesian prior-integration mechanism. The parameter ϕ_t serves as weight for how strongly the priors discount the current similarity update function. Lower values (e.g., near zero) imply that the prior can fully discount the update, if a dimension node does not predict the currently observed outcome P, which reduces the

update to 0 at this node. A larger valie of ϕ_t (e.g., near 1) indicates that the similarity function is integrated into the novel representation more strongly in this case.

The parameter γ_t is defined as based on an individual's γ, estimated during fitting, integrated with the reward hypothesis v_t. Formally,

$$\gamma_t = \gamma + (1 + \exp(-v_t))^{-1} - .5 \tag{12}$$

The exponential transform renders larger values with increasing v_t. Since v_t increases for omitted gains and decreases with a current reward, this means, that the former (no reward) broadens the similarity update and the latter (reward) narrows it, but over time moderated by the accumulated gains, which will eventually approximate a ceiling of 1 due to the exponential transform. The offset of -.5 ensures that the formula is anchored on γ in trial one, since if $v_t = 0$ its exponential transform will be .5. Hence, subtracting .5 yields 0 in trial one. This is the same definition as in Schlegelmilch et al. (2023) that successfully predicts behavior in probabilistic reward learning and risky gambles, including the further implementations of v_t below. In other category learning tasks in which reward is absent in general ($r_t = 0$ in any case), the function reduces to γ, as in the original CAL version (Schlegelmilch et al., 2022).

The parameter ϕ_t is defined as based on an individual's modulation strength ω (estimated during fitting), which can be seen as an individual's error sensitivity for rule errors, which also governs the modulation updates below. Again, we also integrate the reward hypothesis v_t. Formally,

$$\phi_t = .5 \cdot 1/(1 + \exp(-(\omega + v_t)))^{-1} \tag{13}$$

With lower error sensitivity (negative values of ω) the parameter ϕ_t becomes close to zero in general, thus, rather neglecting error feedback during the rule update but fully integrating its Simple Rule priors, becoming a success-driven learning mechanism. Adding to this, lower values of v_t (e.g., current reward obtained) also decrease ϕ_t compared to omitting rewards, which means that the latter loosens the priors to some degree but without strong extrapolation of a novel hypothesis due to the update also becoming broader via γ_t. The integration of both ω and v_t also means, with extremely

low baseline sensitivity to errors (large negative ω) then omitting rewards will hardly contribute anything on top of that.

Note, that CAL, via its modulation (see below) has a specific mechanism to deal with Simple Rule errors, also integrating v_t (stronger modulation in case of errors), routing reactions to obtained and omitted rewards to different cognitive processes. Correspondingly, we consider both of CAL's causal directions of v_t during the fMRI analyses in terms of the direction of neural correlations (e.g., higher activation in reward trials $\sim$ success-driven with strong prior integration vs. higher activation in no-reward trials $\sim$ error-driven modulation updates).

Contrasting. In addition to excitatory generalization CAL abstracts that the currently observed stimulus feature values associate to currently unobserved outcomes via contrasting. Formally, the corresponding inverse generalization update $\Delta w_{mi\bar{P}}$ forms associations between the dimension nodes and the *absent* category ($\bar{P}$):

$$\Delta w_{mi\bar{p}} = \Omega_m\Big(1 - \exp\Big(-\frac{|\sigma^S_{mI} - \sigma_{mi}|^2}{2 \cdot \exp(\gamma_t)^2}\Big)\Big)\Big(\phi_t + \exp\Big(-\frac{w_{miP}}{\sum_{\bar{p}} w_{mi\bar{p}}}\Big)\Big) \tag{14}$$

The contrasting update is stronger on dimension nodes that predicted the absent category (i.e., the same prior weight as in Equation 11, but inverted). Otherwise the formula is conceptually identical to that of excitatory generalization.

Both generalization and contrasting are in strength weighted by Ω_m, which varies between the dimensions. Formally,

$$\Omega_m = (1.1 - \beta_{n=m})\alpha_m \cdot \exp\Big(\sum_n \alpha_m \beta_n v_{mnJPP}\Big) \tag{15}$$

The modulator diagnosticity β_n (here with $n = m$; initialized with $1/M$) informs whether a dimension is currently used as modulator. If so (e.g., $\beta_1 = 1$), then the term applies the cognitive constraint that this dimension will hardly be considered in becoming a Simple Rule, by reducing the update in general. Furthermore, the rule attention this dimension receives for predicting different outcomes (α_m) also moderates the update, such that it reduces for subjectively non-diagnostic rules (i.e., rather enhancing already diagnostic rules). The exponential term takes the gating node (modulator) that links the outcome-response association for category P of the currently

active modulator-rule association as input, which is negative if the current rule is modulated in its prediction (i.e., inverted rule). That is, for modulation to be meaningful (see below), the rule must be left untouched if its errors can be explained by varying contexts, otherwise modulation of it becomes futile. In this case the exponential term becomes close to zero, which represents systematic error discounting, that is, happening if Simple Rule errors systematically repeat in the same context(s), as in the current RB task.

Re-normalization. After adding each update, we re-normalize the simple rule associations via

$$w_{mio} = (w_{mio}^{old} + \Delta w_{mio})/\max(w_{mio}^{old} + \Delta w_{mio}) \tag{16}$$

We then also cap the range at .999 and .001, mainly to allow CAL to learn nothing when the generalization gradient γ becomes very broad (see Schlegelmilch et al., 2022 for further explanations and theoretical implications).

Learning modulation. Learning about potential modulators n is achieved by registering the successes and failures of each simple rule m (with $m \neq n$) on the modulator score v_{mnjok}. The score is updated for the active input J, to associate the category o predicted by the simple rule to the final response category k, further generalizing to adjacent nodes j via similarity (γ_t as above).

For the matching outcome-response gates ($o = k$) the update is:

$$\Delta v_{mnj(o=k)} = T \cdot \exp\Big(-\frac{|\sigma_{nJ}^{S} - \sigma_{nj}|^2}{2 \cdot \exp(\gamma_t)^2}\Big) \cdot (1.1 - \alpha_{m=n})\beta_n \cdot \Big(5 - T' \cdot v_{mnj(o=k)}\Big) \tag{17}$$

For the mis-matching outcome-response gates ($o \neq k$) the update is the same but the direction is reversed, indicated by the sign changes:

$$\Delta v_{mnj(o\neq k)} = -T \cdot \exp\Big(-\frac{|\sigma_{nJ}^{S} - \sigma_{nj}|^2}{2 \cdot \exp(\gamma)^2}\Big) \cdot (1.1 - \alpha_{m=n})\beta_n \cdot \Big(5 + T' \cdot v_{mnj(o\neq k)}\Big) \tag{18}$$

The parameters α_m and β_n act as cognitive constraints as in the previous updates. If the current modulator dimension already carries a subjectively diagnostic Simple Rule (e.g.,$\alpha_m = 1$) it will hardly be considered for becoming a modulator. Furthermore, the update is stronger for modulators already considered as being subjectively diagnostic (e.g., if $\beta_n = 1$). The parameter T' is a teaching signal, which becomes -1 if the simple

rule predicted the wrong outcome, but 1 otherwise. The parameter T is a combination of T' and the free parameter ω_t, which governs the strength of the update. Formally,

$$T = \frac{T'}{(1 + \exp(-\omega_t))} \tag{19}$$

The parameter ω_t, reflecting error sensitivity (modulation strength), is as well affected by the currently obtained reward, with

$$\omega_t = \omega + v_t \tag{20}$$

which is the same definition as for ϕ_t applied as prior weight in the Simple Rule updates, but twice the size for scaling to the different cognitive process. Increasing v_t (reward omitted) leads to increased modulation strength ω_t, while omitting current gains (lower v_t), decreases modulation strength. This can be interpreted in terms of enhanced search for what caused a Simple Rule error or success, if this led to omitting a reward, but to rather retain the current causal representation if the current choice was rewarding.

Modulation error reset. In the original CAL version (Schlegelmilch et al. 2022), we included mechanism that allow CAL to give up on finding the modulated (disjunctive) rules, which is reasonable if participants have no knowledge whether this solution actually exists. However, since participants knew in the current RB task that a disjunctive rule exists, we reduced the original CAL rule-switching definitions to the following. CAL resets the learned associations of single modulator when they lead to a strong prediction error, in order to try finding new/better modulators that will not produce such strong errors. The strength of a modulation error is defined as $1 - 1/(1 + \exp(\sum_m z_{mIP})))$. If the error is larger than $\theta = .8$, CAL counts this as strong error and resets the currently most diagnostic modulator $\max(\beta_{n(n \neq m)})$ back to its initialization state.

Attention learning. For updating α_m (i.e. after the above updates) CAL screens the variation in the category evidence of a dimension m across its nodes i (i.e., r_{miP}), by taking the standard deviations SD_m of the vector of the evidence ratios:

$$\alpha_m = \frac{\text{SD}_m(r_{miP})}{\sum_m \text{SD}_m(r_{miP})} \tag{21}$$

After this, α_m is averaged with the previous α_m ($\sum \alpha_m = 1$).

The update for the modulator diagnosticity, β_n, is defined in a similar manner to that of α_m; in this case using the associations between the rule modulator n on its j nodes and each dimension m separately (gray lines in Figure **??**). Formally,

$$\beta'_n = \sum_{m \neq n} \alpha_m \cdot \mathrm{SD}\Big(\sum_{ok} v_{mnj(o=k)} - \sum_{ok} v_{mnj(o \neq k)}\Big) \tag{22}$$

For each dimension m, the sum of associations on mis-matching gates ($o \neq k$) is subtracted from the sum scores of matching gates ($o = k$) on each j modulator node. A single j score will become positive without re-gating (i.e., $o = k$ has positive and $o \neq k$ has negative associations), but negative with re-gating (i.e., $o = k$ has negative and $o \neq k$ has positive associations). Thus, if the scores strongly vary over the j nodes, there is contextual modulation and the standard deviation (SD) of these nodes will increase. Weighting by α_m (i.e., the value before applying Equation 21) leads to neglecting modulation of non-diagnostic rules. The summed SD's are then normalized:

$$\beta_n = \frac{\beta'_n}{\sum_n \beta'_n} \tag{23}$$

The β_n is then averaged with the previous β_n ($\sum \beta_n = 1$).

Updating of Two Stimuli (Novel Hypothesis)

As described initially, CAL, in the beginning of each trial 'choses' one of the two presented stimuli (e.g., left) for estimating a higher reward-category probability than for the other stimulus. If this choice turns out to be correct (i.e., chosen = rewarded), all updates are applied as described above. However, as the eye-tracking data indicated, if participants chose a stimulus that was not rewarded, then they first processed the chosen stimulus and subsequently the unchosen stimulus. To implement a corresponding process assumption, we added the following hypothesis to the model in case CAL also chose a non-rewarded stimulus.

In brief, all updates above are applied as is for the chosen stimulus first, but omitting the step of contrasting during the Simple Rule update. However, all updates then are repeated for the unchosen stimulus, again omitting the contrasting step, while

re-equating them based on the visual input of the unchosen stimulus. Omitting contrasting in both updates means, that instead of imagining that ‘distinct’ features lead to other outcomes for a given stimulus, the update now appreciates the ‘actually’ distinct stimulus features. That is, applying excitatory generalization to the left and right stimulus will only lead to rule extrapolation like for contrasting, if both stimuli actually differ on a feature dimension. However, the resulting Simple Rule update will be weaker than in reward trials if ρ^{I} is estimated to be larger than zero (i.e., the reduced udpate strength does not ‘add up’ when applied to each stimulus sequentially, because contrasting is removed). Similarly, since modulation would be potentially applied to the same modulator nodes (e.g., wings = one circle) the modulation update would double in strength, which seems undesirable, as we would generally assume that splitting attention between two stimuli decreases their respective processing depth to some degree. Therefore, we prevented double-strength updates during modulation learning by dividing the whole update (Equations 17 and 18) by 2 in this case.

Solving RB and Self-Supervised Learning (Novel)

As discussed in the main manuscript, after solving the RB task, participants were hardly affected by no-reward feedback. We interpret this as ignoring no-reward feedback as corroborated by the eye-tracking data. For this, we implemented the hypothesis in CAL, that once it considered the task as being solved, it takes its very own predictions as teaching signals. This means, after this time point, it treats every of its own decisions as correct/rewarding, becoming a self-supervised learning model. This also means, the above specification of processing only the chosen stimulus applies in all trials after CAL considered the task as solved, in a self-reinforcing manner.

However, the question is how to reach at a reliable impression *when* the task can be viewed as solved. For this, we reasoned that participants counted how often their predictions are correct in a row. In fact, this in itself is not a novel hypothesis, but also helps explaining observed phenomena in other probabilistic reward learning tasks and risky gambles (e.g., Gamblers fallacy; see Schlegelmilch et al., 2023). However, the novel

assumption is, that they use this count here to decide when the task is considered to be solved. For this, we used the current outcome prediction strength $\sum z_{mIP}$, and assumed that participants count the number of trials in which this prediction is highly confident and correct (i.e., expected probabilities exceeding a 80% threshold). If this count reaches three consecutive correct decisions, CAL, in the next trial, begins with self-supervised learning.

Thus, reaching this confidence is influenced by CAL's rule learning parameters, and random trial-and-feedback order. That is, while CAL can occasionally develop strong predictions early on, spurious probabilistic feedback might falsify these predictions before reaching three consecutively correct and confident choices. In other cases, however, it can be that CAL receives eight trials with actually true feedback by chance, but only reaches a confident category representation after about five of them. In this case, while CAL might develop correct predictions quite quickly, but becoming confident only after seeing more correct trials, to then considered the task as being solved. Note, that we assume a different process in the U task, as we instructed participants to use a different strategy to solve the task (i.e., memorization), as explained in the main manuscript.

Configural Memory

Finally, a memory update strengthens the association h_{SP} between the memory representation of stimulus S and its paired category P:

$$\Delta h_{SP} = \frac{B}{1 + \exp\Big(- \lambda_t + \exp(1 + 1/(F \cdot C) \sum_{sk} h_{sk}^{\text{old}})\Big)} \tag{24}$$

This equation is applied to both presented stimuli equally. First, the parameter λ_t represents reward and stimulus-specific encoding strength, which we estimate in fitting. Second, the values of Δh_{SP} can range between 0 and B, which is defined as $B = 1/M \cdot (C - 1)$, with M number of dimensions, and C number of categories (i.e., assuming that a higher number of features and categories are more difficult to memorize, which however is a constant value in the given task). The term $\exp(1 + 1/(F \cdot C) \sum_{sk} h_{sk}^{\text{old}})$ represents the average of associative strengths of existing

memories (with F number of instances with non-zero associations). Adding this term implements a decelerated learning function, annealing the strengths of all updates over time, as theoretically discussed in the main manuscript.

Regarding the parameter λ_t, we estimate trial and stimulus-specific update strengths. As highlighted in the main manuscript, if CAL obtains a current reward, then the chosen stimulus is updated with $\lambda_t = \lambda + \lambda^{Reward}$ and the unchosen stimulus with $\lambda_t = \lambda - \lambda^{Reward}$. In no-reward trials, the chosen stimulus is updated with $\lambda_t = \lambda - \lambda^{NoReward}$ and the unchosen stimulus with $\lambda_t = \lambda + \lambda^{NoReward}$. Thus, we assume that the average update strength in all trials is equal to the λ estimate, but diminishing in general due to increased memory load over time. Moreover, this implementation allows directly comparing whether λ^{Reward} differs in sign and/or strength from $\lambda^{NoReward}$. For one, as the eye-tracking data suggested in the U task, in which these parameters are theoretically central, the chosen stimulus if rewarded received more attention than the unchosen stimulus (i.e., expected $\lambda^{Reward} > 0$), but also that the chosen stimulus if rewarded received more attention than the unchosen stimulus in error trials (i.e., expected $\lambda^{Reward} > \lambda^{NoReward}$). Thus, we test the corresponding hypotheses via model estimation in the main manuscript via direct comparison, but also via correlating the obtained parameters with the eye-tracking data, also serving as cross-validation of the CAL estimates in the U task.

Interpretation of CAL predictors

In the main manuscript we highlighted the use of four predictors used in choice and outcome processing for RB and U, respectively. While the choice predictors are straight forward (trust in rule solution in RB, memory-derived reward probability in U), the interpretation of the outcome predictors deserves further notice, especially in RB. These predictors are illustrated in Figure 6. Generally, before the solution the signal is higher in Reward than No Reward trials. However, after the solution the signal is basically equal between both trials due to self-supervised learning, and generally low in RB, and intermediate to high in U. In U, this due to continued Hebbian learning, while

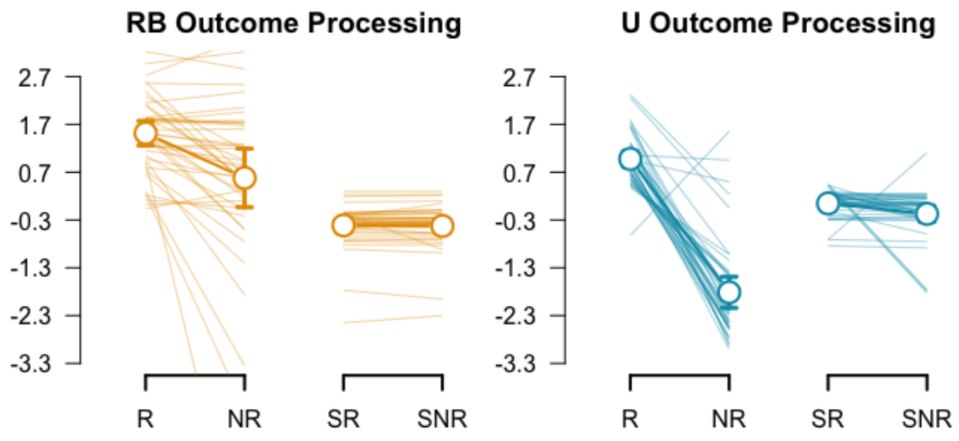

Figure 6. *CAL Outcome Processing Derivates by Trial Type. R= Reward, NR= No Reward trials before CAL switched to self-supervised learning. SR= Reward, SNR= No Reward trials after CAL switched to unsupervised learning. Thin lines = individual estimates, thick = average + 95% CI. See text for explanation and interpretation.*

in RB, the low values reflect CAL's reward function which reflects generally increased cognitive control (modulation) and reduced rule extrapolation (Simple Rule updates), further explained below.

Regarding RB, we used v_t as calculated for processing Simple Rule updates based in the chosen stimulus (i.e., as calculated in Eq. 12) but inverted by its sign to let larger values reflect positive reward signals and smaller values no-reward signals. That is, positive reward signals translate to more precise Simple Rule updates compared to no-reward trials (i.e., narrower generalization function based on γ_t). While CAL only processes the chosen stimulus in reward trials, however, it processes the chosen and unchosen stimuli in no-reward trials. Furthermore, in no-reward trials CAL's feature contrasting (Eq. 14) is replaced by generalization based on both stimuli (i.e., Eq. 11 applied to chosen then unchosen), thus, reflecting a comparison of both stimuli regarding which features may form a Simple Rule. Thus, processing both stimuli in itself does not imply doubles update strengths, therefore not affecting the interpretation of v_t outlined above. Regarding Simple Rules then, finding positive correlations with fMRI data would indicate stronger rule generation in reward compared to no-reward trials, further declining in overall strength after solving the task, due to CAL's decaying

v_t and self-supervised learning (treating all feedback as rewarding/correct).

Negative correlations of v_t, can be interpreted as stronger influence of error processing in no-reward trials, or negative reward signals. Thus, besides perhaps higher salience or errors or affective reactions to them, the functional interpretation would concern CAL's error-driven learning, which only takes place in its modulation mechanism, designed to structure the problem based on Simple Rule errors. We therefore would intepret negative correlations of v_t as related to higher-order structuring processes or search for *conditional* hypotheses. Again, note, we assumed that, in no-reward trials, CAL's split attention to both stimuli. In modulation, we therefore reduced the overall strength of modulation updates by dividing the effective update strength by 2, that is, after taking reward vs. no-reward variations in v_t into account. Thus, applying modulation to both stimuli does not mean that the resulting processing strength becomes additively stronger relative to reward trials, but it becomes stronger due to the influence of v_t. Thus, interpreting v_t in terms of error-processing is straight forward in any case. Given that the described hypotheses well predict several central details of the individuals' behavioral and eye-tracking data, we believe that a functional interpretation in terms of searching for conditional hypotheses in cases of errors is reasonable.

Crucially, since we freely estimated reward vs. no-reward signals for updating the chosen stimulus in U (which we took as fMRI predictor), the basic reward signal interpretations are the same as for RB (higher values reflect positive reward signals). Thus, the conjunction analysis regarding U and RB should at least reveal those areas which are processing reward signals. Indeed, this seemed to be the N.accumbens. Exclusive mapping in each task then should reveal areas more relevant for each learning strategy, to which we would apply the functional interpretations of the above measures.

Figure 7 shows an example participant, bringing together choices, response times, and CAL's model predictors in each task. The top row shows the participants choices (orange line; left vs. right stimulus) and CAL's fitted choice probability (black line). The colored symbols at the top indicate the participant's canonical choice accuracy

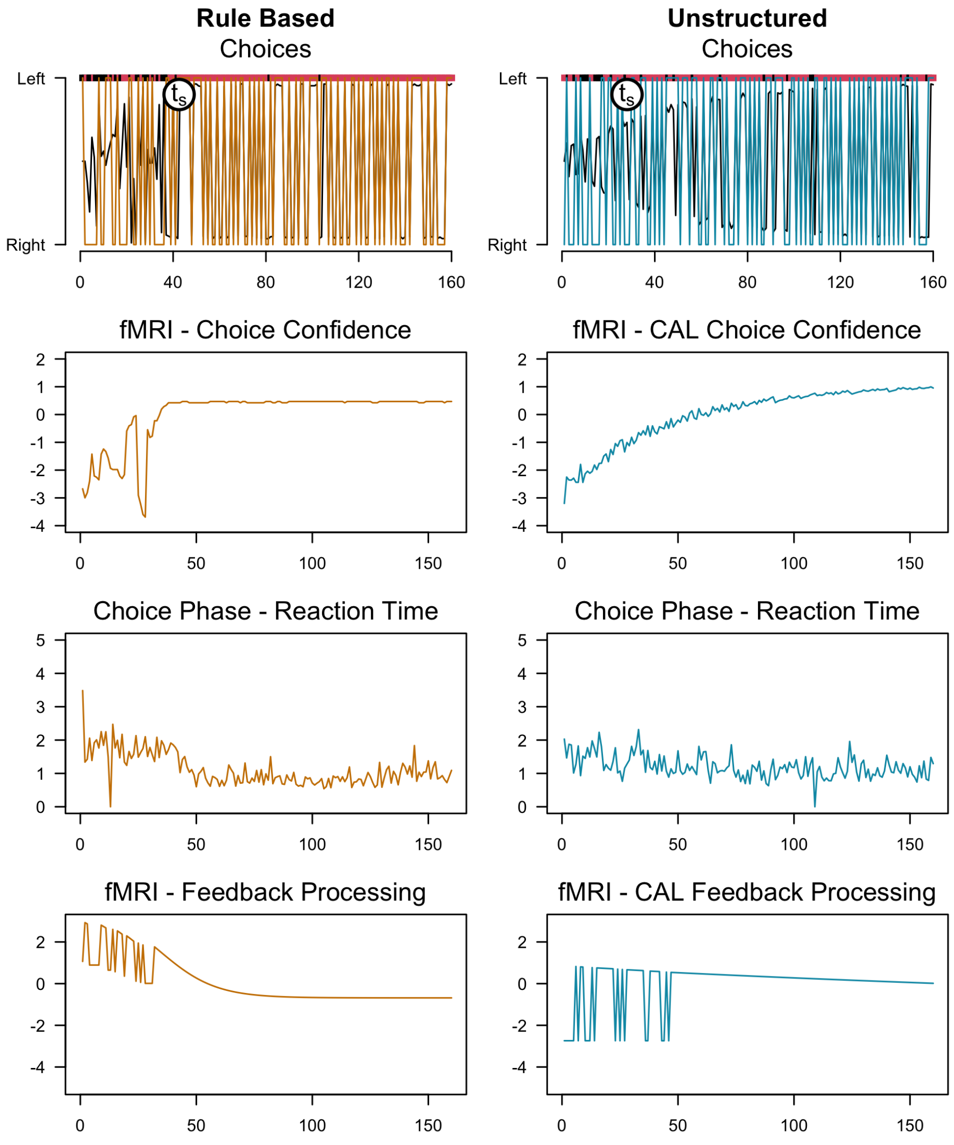

Figure 7. *Example Participant 59. See text for description*

(black = error, red = correct). The second row (CAL Choice Confidence) shows the derived CAL-informed predictors in the fMRI analyses. For comparison, the third row shows the response times (choice phase). Finally the fourth row shows CAL's feedback processing strength. Before finding the solution, trial-wise variations depend on the reward obtained by CAL (higher values = reward). Over training, the accumulated reward moderates CAL's RB feedback strength in RB (generally stronger [self-confirmatory] rule generation in later trials, and generally weaker context-searching modulation in late trials). In U, high values reflect stronger encoding of the chosen

stimulus in reward trials, and lower values reflect weaker encoding of the chosen stimulus in no-reward trials. Over training, the overall strength reduces depending on how much has been stored in memory already (the more has been stored, the weaker the subsequent updates). Generally, after the solution is found, every trial is treated as a reward trial due to CAL's feedback re-evaluation.

Bibliography

Glaholt, M. G., & Reingold, E. M. (2011). Eye movement monitoring as a process tracing methodology in decision making research. *Journal of Neuroscience, Psychology, and Economics*, *4*(2), 125. doi: 10.1037/a0020692

Shimojo, S., Simion, C., Shimojo, E., & Scheier, C. (2003). Gaze bias both reflects and influences preference. *Nature neuroscience*, *6*(12), 1317–1322. doi: 10.1038/nn1150
